# Political Trust Influences the Relationship Between Income and Life Satisfaction in Europe: Differential Associations With Trust at National, Community, and Individual Level

**DOI:** 10.3389/fpubh.2021.629118

**Published:** 2021-03-15

**Authors:** Jocelyne Clench-Aas, Arne Holte

**Affiliations:** ^1^Division of Mental and Physical Health, Norwegian Institute of Public Health, Oslo, Norway; ^2^Department of Psychology, University of Oslo, Oslo, Norway

**Keywords:** well-being, income, trust, satisfaction, moderation, Europe

## Abstract

**Background:** A high level of well-being is associated with personal, community and national income, as well as personal, social and political trust. How these measures relate to each other within and between countries and within and across structural levels of society is largely unknown. To study this, we propose a three-layer nested socio-structural model. Each layer (individual, community, country) contains a measure of income, trust and satisfaction.

**Method:** With this model, we analyzed data from two waves of the European Social Survey (ESS, 2006, 2012) in 19 countries (*N* = 72,461; weighted *N* = 73,307) with multilevel techniques. Indicators were personal, community, and national income; personal, social and political trust; and personal life satisfaction, social and political satisfaction.

**Results:** Personal life satisfaction was associated with all income and trust variables. Greatest effect on personal life satisfaction, came from the national level, including political trust and income. However, 2/3 of the variance in personal life satisfaction came from income, that is personal, community and national. Within each socio-structural level, satisfaction was associated with income, but significantly modified by trust. When income and trust at all three levels were included, there was a significant association of the national layer on the social layer, and of the social layer on the individual layer as to the income–personal life satisfaction relationship. Consistent with the “the buffer hypothesis,” all three forms of trust acted as a buffer against the effect of personal income on life satisfaction. Low-trust countries had strong income–personal life satisfaction associations and the moderating role of trust was also stronger. High- and medium-trust countries had no such associations. Likewise, direct associations between political and personal trust were much stronger in the low-trust countries.

**Conclusion:** The model presented in this study provides authorities with a framework for policies that will improve the general well-being of their population. Trust and income strongly influence personal life satisfaction. Money is the most important. However, trust forcefully dampens the effect of income. Politicians who want to enhance their population's personal life satisfaction, should raise the levels of trust in their electorate.

## Introduction

Most people would, without doubt, prefer to live in a society with a high level of trust rather than in one with a low level (1). This concerns personal trust in terms of self-confidence and self-esteem. It concerns social trust in terms of trusting other people. It concerns political trust in terms having trust in how the country is run.

One important effect of trust is that it influences a person's well-being (1). Well-being refers partly, to an individual's long term, cognitive evaluation of one's life as a whole, life satisfaction, and partly to a more short-term, positive emotional or affective state, happiness (2–4). Life satisfaction may be divided into different facets: (1) satisfaction with one's personal life, personal life satisfaction; (2) satisfaction with one's social environment, social satisfaction; and (3) satisfaction with how the country is run, political satisfaction.

Furthermore, income seems to influence both personal, social and political trust as well as the different facets of satisfaction. This applies to personal income, community income as well as national income (5–15). For a more detailed review of studies on relationships between well-being, income and trust, see Supplementary Material 1.

However, two limitations to most of these studies are that they only show bivariate associations and that they rarely account for more than one or two levels of society, i.e., individual, community or national. Consequently, the relative contributions of personal, community and national income, and personal, social and political trust on the different facets of satisfaction with life is still unknown.

Examples of unanswered questions are: Which one of personal, community or national income matters the most to which facets of life satisfaction (6, 10, 16–18)? What are the roles of personal, social and political trust within these relationships? May trust play a moderating role on the relationships between income and satisfaction? May the different facets of trust (e.g., personal, social and political), have separate relative associations at different structural levels of a society (e.g., individual, community, and national)? May they have separate moderating effects on well-being, social and political satisfaction, and, respectively?

In 1991 Göran Dahlgren and Margaret Whitehead introduced a holistic multilevel model, the so called “rainbow model,” to conceptualize how economic, environmental and social inequalities may determine people's risk of getting ill, their ability to prevent sickness, or their access to effective treatments (19). They placed the individual at the center of the model, with its fixed factors such as sex, age, and constitutional endowment. Surrounding them were different layers of modifiable factors that can influence health, such as individual lifestyles; social and community networks, economic, and cultural and physical environment. This framework has inspired researchers to construct a range of hypotheses about the determinants of health and to explore their relative influence on different health outcomes.

To be able to determine the relative associations between personal, community and national income and personal, social and political trust, and personal satisfaction with life, and to determine the possible moderating role of trust on the relationships between income and different facets of satisfaction, we designed a similar model.

Like Dahlgren and Whitehead's model of social determinants of health (19), we regard the society as a construction with three nested socio-structural layers. The main determinants of well-being are layered from the individual to the major structural environment and each layer can influence the well-being of the individual. We define the individual person as the basic unit (micro layer). We then regard the individual as nested into her or his local community (mezzo layer), which again is nested into the country (macro layer). Likewise, we regard the individual person's economy as nested into the economy of her or his local community, which again is nested into the economy of the country.

**Figure 1:**
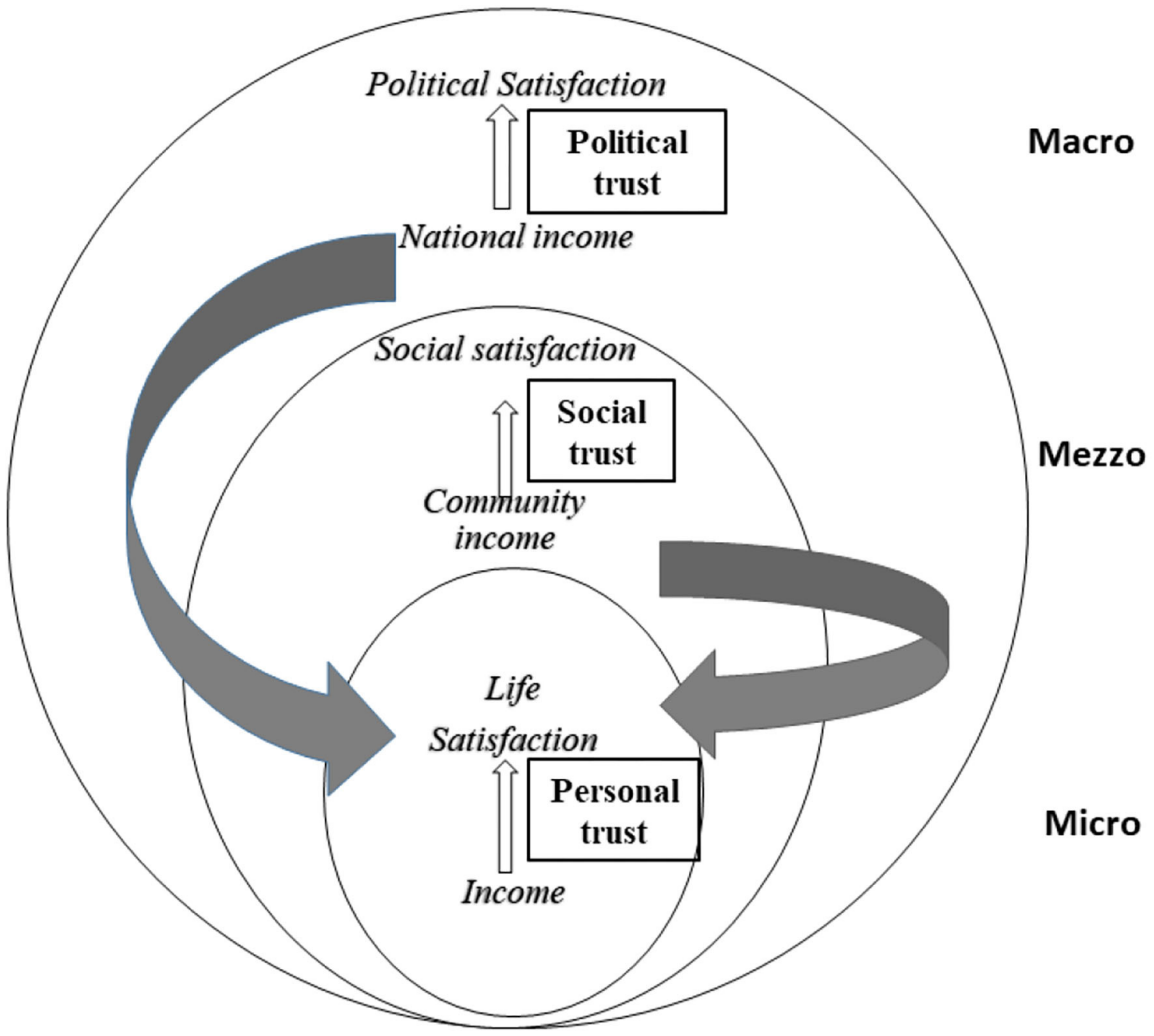
A schematic presentation of the socio-structural model, indicating the three layers. Micro level = Individual. Mezzo level = Community. Macro level = Country. Indicators of income: Micro level = Personal income. Mezzo level = Community income. Macro level = National income. Indicators of satisfaction: Micro level: Personal life satisfaction (PLS). Mezzo level = Social satisfaction. Macro level = Political satisfaction.

We then hypothesize that there is a direct association between income, trust, and satisfaction within each layer. In addition, we hypothesize that trust, i.e., personal trust, social trust, and political trust, modifies the associations between income and satisfaction (Figure 1). In particular, we hypothesize that personal, social and political trust acts as a buffer against the effect of personal income on personal life satisfaction (“the buffer hypothesis”).

Why then is it important to investigate the effects of income and trust on satisfaction at all three levels of a society, individual, community, and country? Understanding of how these parameters relate to each other may help us getting a deeper comprehension of how societies work. Good policy development in terms of getting a population who is satisfied with life, their social environment, and how the country is run, may depend on which of these parameters have the greatest positive effects on their well-being.

But, how can including trust shed light on the multilevel relationship between well-being and income? Until we have analyzed these associations together in one and the same model, taking into account effects from all three layers of society, there may be difficulties in interpreting consequences of the single relationships.

Why then could investigating the moderating role of trust in the relationship between well-being and income at the three different layers be helpful? Reaching a better comprehension of the role that different kinds of trust play in moderating effects of income on satisfaction, may be crucial in understanding basic mechanisms of society. This may again be decisive in determining which political strategies should be approached in aiming to enhance people's well-being and health. Imagine for example, that if in some countries, enhancing social trust, makes life simpler, easier, more pleasant, and friendly, this may be as effective a measure to enhance well-being and health as is increasing personal, community or national income.

Building on previous findings, we take the field one step further by integrating income, trust, and satisfaction into one holistic three-level nested model as described above, to answer the following research questions (RQ):

**RQ1** What is the statistical contribution of income and trust at each socio-structural layer, i.e., micro (individual), mezzo (community), macro (country), to variation in their respective satisfaction parameter, i.e., personal life satisfaction, social satisfaction, and political satisfaction (“Within layers effects”)?

**RQ2** What is the statistical contribution of each of the individual parameters within each socio-structural layer to variations in personal life satisfaction (“Total holistic effect”)?

**RQ3** What is the relative, independent and simultaneous contribution (effect size) of each content theme at each socio-structural layer to variation in personal life satisfaction (“Effect size of holistic approach”)?

**RQ4** What is the relative contribution (effect size) of each socio-structural layer to variation in personal life satisfaction (“Between layers effects”)?

**RQ5** What is the relative contribution (effect size) of each content theme, i.e., income and trust to variation in personal life satisfaction (“The themes effects”)?

**RQ6** Does the relationship between income and well-being differ between countries according to their level of trust (“Variation by countries”)?

**RQ7** Does trust function as a moderator, in addition to its direct effect, either within the layers or in the holistic model? (“The buffer hypothesis”)?

## Materials and Methods

Supplementary Material 2 provides a more detailed description of methods used in this study.

We used data from the European Social Survey (ESS). Data are highly comparable across nations, with a high response rate in all rounds.

In the present study, the data were restricted to the years complete with respect to the choice of variables. Thus, we used the cumulative dataset for rounds three and six (corresponding to 2006–2012), found on ESS web page (www.europeansocialsurvey.org). Data from the respondents in the 19 countries that participated in both rounds and included the variables of interest, were used. The final sample was *N* = 72,461 (Weighted-*N* = 73,307) and had a mean age of 48 years and 54% females (in the weighted sample 46 years and 51%. The data are freely available on the European Social Survey internet site (https://www.europeansocialsurvey.org/).

### Measures

#### Level Defining Variables

The primary analysis is multilevel. In multilevel analysis, levels are specified prior to the analysis that defines the clusters that the analyses are performed within. For this study, we used three levels of analysis, (1) micro (individual), (2) mezzo (community), and (3) macro (country). See Supplementary Material 2—Methods for more details in definitions of variables.

*Micro level* was defined by the informant's personal number.

*Mezzo level* was defined by two nested variables: (a) regions within each country and (b) social class. The respondent's social class was determined using education and occupation See more details concerning these two variables in Supplementary Material 2–Methods. The value is a mean of the respondent and his/her partner if present. If data on occupation or education was missing for the partner, we used the respondent's education or occupation.

*Macro level* was defined by 19 countries: Belgium (BE), Bulgaria (BG), Cyprus (CY), Denmark (DK), Finland (FI), France (FR), Germany (DE), Ireland (IE), Netherlands (NL), Norway (NO), Poland (PL), Portugal (PT), Russia (RU), Spain (ES), Slovakia (SK), Slovenia (SI), Sweden (SE), Switzerland (CH), and United Kingdom (GB).

#### Income

Three income variables were used as independent variables.

At the *micro level*, we used personal income. Personal income was measured in terms of the annual household income of the individual. For further information on classification of personal income, and the necessary standardization procedures used to homogenize slightly different methods in classifying personal income, see Supplementary Material 2, Table 2 in Supplementary Material 3.

At the mezzo level, as seen in other studies (20), we used community income. Community income was calculated for this study as the aggregate of the household income value by country, region, and social class. The aggregate value was divided by 1,000 to ease interpretation.

At the *macro level*, we used national income. National income was measured in terms of the Gross Domestic Product (GDP). The unit of measure was GDP per capita, PPP (current international $). Purchasing power parity (PPP) is a way to estimate exchange rates between currencies that account for purchasing power. GDP PPP controls for the different costs of living and price levels enabling a more accurate depiction of the different countries level of production. For the analyses in this study, we used the log of GDP (Ln GDP) per capita divided by 1,000. For further information on classification of national income, see Supplementary Material 2.

#### Satisfaction

Personal life satisfaction was used as an indicator of well-being. Personal life satisfaction was used as the dependent variable, except for research question RQ1 and RQ7, where also social satisfaction and political satisfaction were used as dependent variables.

At the *micro level*, we used personal life satisfaction. Personal life satisfaction was measured by the following item “All things considered, how satisfied are you with your life as a whole nowadays?” Responses were given on an 11-point scale ranging from 0 to 10, 0 = “extremely dissatisfied,” 10 = “extremely satisfied” (21).

At the *mezzo level*, we used social satisfaction. Social satisfaction was measured by a variable constructed as the average of the responses to four questions: (1) “Do you feel close to the people in local area?”, with response alternatives ranging from 1 = “Disagree strongly” to 5 = “Agree strongly”; (2) “Do you feel people treat you with respect?”; (3) “Do you feel people in local area help one another?”, both of the last questions ranging in response from 0 = “Not at all” to 6 = “A great deal”; and (4) “Do you feel safe walking alone in local area after dark,” with response alternatives ranging from 1 = “Very unsafe” to 4 = “Very safe,” (Cronbach's alpha = 0.59). These questions cover the areas of belonging, social support and respect, as well as safety in the local area. Questions 1 and 4 were extended to conform to the range of questions 2 and 3 (22). The final variable represented the average of the four questions.

At the *macro level*, we used political satisfaction. Political satisfaction was measured by a variable constructed as the average of the responses to five questions: (1) “How satisfied are you with the present state of the economy in your country?”; (2) “How satisfied are you with the national government?”; (3) How satisfied are you with the way democracy works in your country?”; (4) “How satisfied are you with the state of education in the country nowadays?”; and (5) “How satisfied are you with the state of health services in the country nowadays?,” all with responses given on an 11-point scale ranging from 0 to 10, 0 = “Extremely dissatisfied,” 10 = “Extremely satisfied” (Cronbach's alpha = 0.83). The variable political satisfaction was the sum of the answers to the five questions and ranged from 0 to 50 (22).

#### Trust

We used three variables of trust, both to measure direct effects of trust and to explore trust as potential moderators. These variables were developed for ESS, and have been in use since 2006 (23).

At the *micro level*, we used personal trust. Personal trust was measured by the following item: “In general I feel very positive about myself.” Responses were given on a 5-point scale ranging from “Agree strongly” to “Disagree strongly” (24). The variable was recoded inversely.

At the *mezzo level*, we used social trust. Social trust was measured by the following item “Generally speaking, would you say that most people can be trusted, or that you can't be too careful in dealing with people?” Responses were given on an 11-point scale ranging from 0 to 10 (0 being “You can't be too careful.” and 10 being “Most people can be trusted”) (23, 25). This measure of trust has been observed to be stable and its validity confirmed (26).

At the *macro level*, we used political trust. Political trust was measured by the five following items: “How much do you personally trust the country's parliament?”; “How much do you personally trust the police?”; “How much do you personally trust the legal system?”; “How much do you personally trust the politicians?”; and “How much do you personally trust the political parties?.” Responses to each were given on an 11-point scale ranging from 0 to 10 (0 being “you do not trust an institution at all” and 10 being “you have complete trust”) (23, 25). The answers were added, yielding a parameter with a range of 0–50.

#### Confounders

The demographic variables adjusted for, in all the analyses, were year of investigation, gender, age and age^2^, number of people living regularly as members of household, marital status (dummy variable), education, occupation, being permanently sick or disabled, being unemployed, and mental health. Being permanently sick or disabled and being unemployed were two alternatives in a question concerning main activity last 7 days (dummy variable). Mental health was a combination of two questions concerning feeling depressed or anxious. The two variables were recoded to either being most of the time or all of the time depressed or anxious, as opposed to less than that. The two variables were then combined so that the individual had at least one of the two conditions. Age is well-documented to have a curvilinear relationship, and therefore it is highly recommended to use the squared function (3).

### Stratification of Countries

Each of the 19 countries was ranked according to its level of social and political trust separately. The resulting rankings were added together, and a new ranking performed of the combined value. The countries were then divided equally into three groups, Group 1, exhibiting the highest trust levels, included the Nordic countries of Denmark, Finland, Norway, and Sweden in addition to Switzerland and Netherlands; Group 2, exhibiting a medium-trust level, included United Kingdom, Belgium, Germany, Ireland, France, and Spain; and finally Group 3, exhibiting the lowest trust levels, included Slovenia, Cyprus, Slovakia, Russia, Portugal, Poland, and Bulgaria (See Supplementary Material 3, Table 3).

### Statistical Analysis

The analyses were conducted using the Statistical Package of the Social Sciences (SPSS), version 25.0. All data were weighted in accordance with the ESS guidelines before conducting the analyses (27).

The primary method of analysis was the multilevel analysis. In SPSS this is done with the module Linear Mixed models (28). The data were weighted in these analyses using the post-stratification weight that includes a design weight. A three-level approach was used as the main method of analysis. The levels chosen were (1) the unit of measure is the individual; (2) community, which for practical purposes was defined using two variables, (a) within country region and (b) social class; and (3) country. The outcome variables were personal life satisfaction (all research questions, RQ), social satisfaction (RQ 1 and 7), and political satisfaction (RQ 1 and 7), representing the different hierarchical levels. For each of these layers, investigation year, gender, age and age^2^, number of people in household, marital status, education, occupation, being permanently sick or disabled, being unemployed, and mental health, were entered as covariates. Additionally, separate economic indicators for each layer were used, personal income, community income and national income. Finally, trust variables for each layer were used (i.e., personal trust, social trust, and political trust). Unstandardized beta-coefficients with standard errors, are reported.

Moderation analyses were performed both by introducing an interaction in the multilevel analyses, and by using Andrew F. Hayes' PROCESS tool for SPSS. The latter was unfortunately unable to incorporate multilevel analyses; however, the analyses were performed on the country groups based on overall trust that were slightly more homogenous.

**Figure 2:**
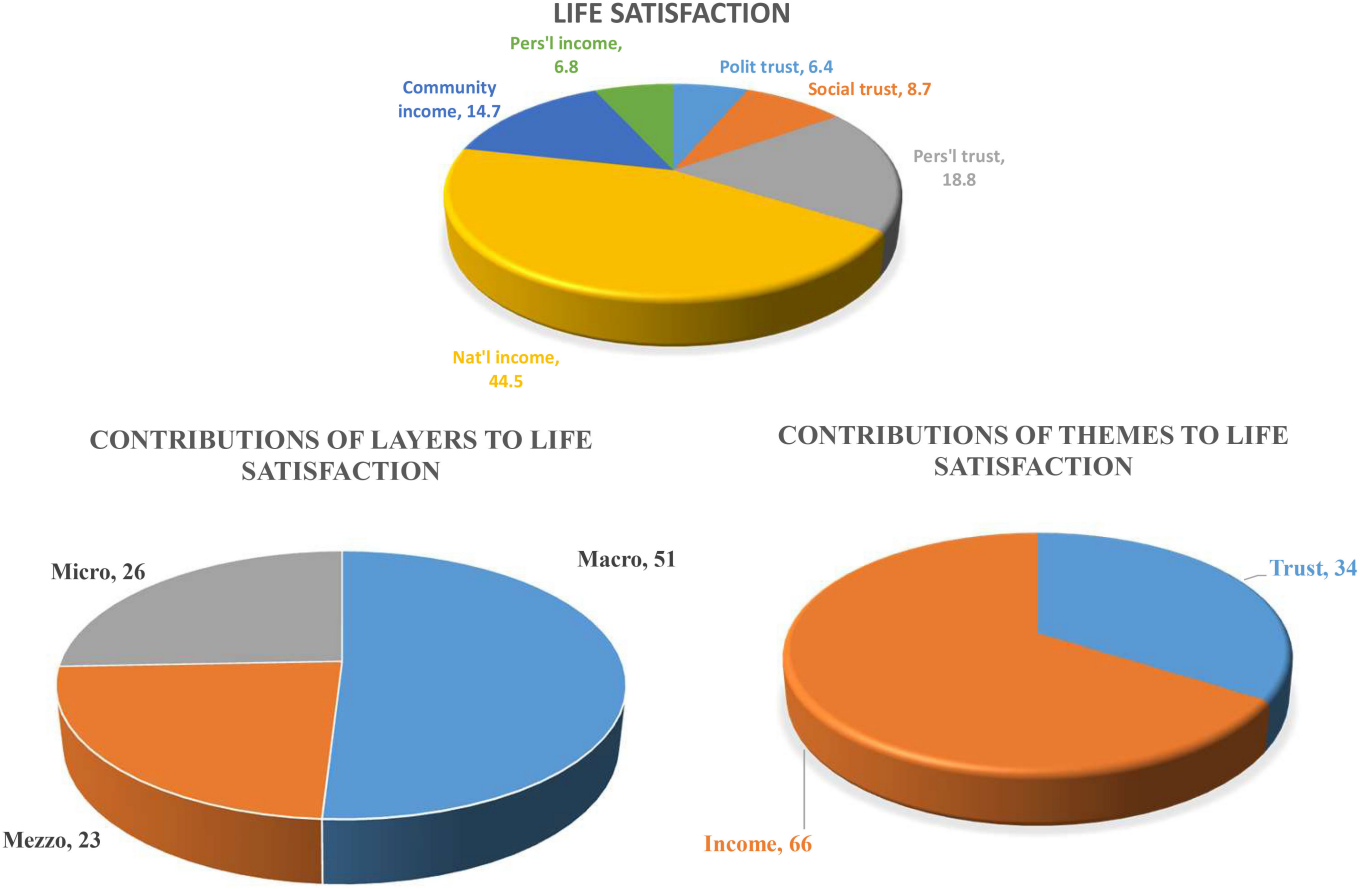
Overview of the relative importance of personal, community, and national income; personal, social and political trust for life satisfaction, by layer and by theme. Results of multilevel analysis. Levels: individual, community, and country. Percent. Valid *N* (listwise) = 44,833. Micro level = Individual. Mezzo level = Community. Macro level = Country. Indicators of income: Micro level = Personal income. Mezzo level = Community income. Macro level = National income. Indicators of satisfaction: Micro level: Personal life satisfaction (PLS). Mezzo level = Social satisfaction. Macro level = Political satisfaction.

Analyses of relative effect was done by multiplying unstandardized coefficients with the population mean of the parameters used. The pie charts in Figure 2 were constructed by multiplying the calculated beta with the weighted average for the entire population for each variable. Missing data were excluded listwise in the regression analysis. For number of missing values see Table 1 in Supplementary Material 3. Model fit was evaluated by significant *R*^2^ in the multiple linear regression.

**Table 1:** Weighted means/ranges of the primary variables of interest in the study by country.

	**PLS**	**Social satisfaction**	**Political satisfaction**	**Personal income euros**	**Community income euros Range**	**National income euros**	**Personal trust**	**Social trust**	**Political trust**	**Regional unit NUTS**
BE	7.4	4.4	6.0	33,662	77,041	39,204	3.7	15.3	24.4	2
CH	8.1	4.7	6.7	72,533	162,918	51,250	4.0	17.7	29.4	2
DE	7.2	4.5	5.0	33,582	85,784	39,111	4.1	15.7	24.2	1
DK	8.5	4.8	6.7	48,834	98,309	42,111	4.0	20.3	32.3	2
ES	7.2	4.6	4.5	24,033	171,591	31,825	4.0	15.2	19.9	2
FI	8.0	4.5	6.8	37,547	76,446	377,835	3.9	19.3	31.0	3
FR	6.5	4.4	4.7	33,455	136,793	36,303	3.5	14.9	21.5	2
GB	7.3	4.2	5.0	31,198	132,857	35,784	3.8	16.8	22.9	1
IE	7.1	4.6	4.9	40,124	113,720	46,923	4.0	17.0	22.4	3
NL	7.8	4.5	5.9	38,916	172,606	44,192	3.8	17.7	27.9	2
NO	7.9	4.9	6.5	67,575	109,959	59,622	3.7	19.7	29.2	2
PL	6.9	4.4	4.1	9,103	79,650	19,708	4.0	12.6	15.9	2
PT	5.8	4.6	3.6	14,049	97,684	26,471	4.0	12.6	16.5	2
SE	7.9	4.7	6.0	42,999	86,079	41,201	4.0	18.8	28.0	3
SI	7.0	4.6	4.4	17,909	59,609	27,190	4.0	14.0	18.0	3
CY	7.2	4.5	5.1	26,845	118,492	31,211	4.0	12.3	21.5	1
BG	4.6	4.3	2.9	3,790	11,543	14,631	4.0	10.8	11.4	3
RU	5.6	4.1	3.8	6,608	14,309	20,187	4.0	13.4	16.1	Other
SK	6.3	4.2	4.5	11,991	35,814	23,565	3.8	12.9	18.0	3

*Weighted N = 73,307. Personal income is measured as yearly household income, community income per thousand is measured aggregated mean of household income for country, region and social class; national income is measured as Ln GDP (PPP) per capita per thousand. Ranges of income: Personal income, −4.45–200; Community income, −3–74; National income, 2.4–4.2; Ranges of trust: Personal trust, 1–9; Social trust, 0–30; Political trust, 0–50. Ranges of satisfaction: Life satisfaction, 0–10; Social satisfaction, 0.3–173.0; Political satisfaction, 0–10*.

### Ethics

The data are available without restrictions, for not-for-profit purposes.

In accordance with the ESS ERIC Statutes (Article 23.3), the ESS ERIC subscribes to the Declaration on Professional Ethics of the International Statistical Institute. The Research Ethics Committee reviews applications for studies for which the ESS ERIC is directly responsible, that is, which it directly contracts.

## Results

### Descriptives

Satisfaction varied greatly between countries, with personal life satisfaction varying from 4.6 in Bulgaria to 8.5 in Denmark; social satisfaction varying from 4.1 in Russia to 4.9 in Norway; and finally political satisfaction varying from 2.9 in Bulgaria to 6.8 in Finland (Table 1).

Also income varied between countries, with personal income varying from 3,790 in Bulgaria to 72,533 in Switzerland; community income varying from 11,543 in Bulgaria to 172,606 euros in Netherlands; and finally national income varying from 14,631 in Bulgaria to 59,622 euros in Norway.

Trust varied too, but with personal trust being fairly uniform varying from 3.5 in France to 4.1 in Denmark; social trust varying from 10.8 in Bulgaria to 20.3 in Denmark; and finally, political trust varying from 11.4 in Bulgaria to 32.3 in Denmark.

Intercorrelations between the variables are presented in Supplementary Material 3, Table 4.

### Layers and Themes

Answers to **RQ1** (“Within layers effects”) are shown in Table 2. There was a significant and positive contribution of all three income variables on their respective satisfaction parameter within each layer (model 1). For personal life satisfaction, the association with personal income was strengthened upon addition of the personal trust parameter. For social satisfaction, upon the addition of social trust, the positive association between social satisfaction and community income was changed to a significant negative association. For political satisfaction, the association was substantially weakened, but still significant (model 2).

**Table 2:** Results [Beta (SE)] of multilevel analysis of personal life satisfaction (micro level), social satisfaction (mezzo level) and political satisfaction (macro level).

	**Level**	**Micro**	**Mezzo**	**Macro**
**Model**	**Measure of satisfaction**	**Personal life satisfaction**	**Social satisfaction**	**Political satisfaction**
	**(Mean/SE/Sig)**			
1	National income			2.396 (0.050)^***^
	Community income		0.005 (0.000)^***^	
	Personal Income	0.010 (0.000)^***^		
	Individual level variance—within	3.560/3.206	0.777/0.720	2.110/2.051
	Three level variance—between	1.353/0.793	0.099/0.066	1.524/0.532
	Pseudo *R*^2^ within	0.105	0.042	0.032
	Pseudo *R*^2^ between	0.371	0.282	0.592
2	National income			0.885 (0.047)^***^
	Community income		−0.001 (0.001)^*^	
	Personal income	0.016 (0.002)^***^		
	Political trust			0.067 (0.006)^***^
	Social trust		0.042 (0.001)^***^	
	Personal trust	0.565 (0.017)^***^		
	Interaction trust × income	−0.002 (0.000)^***^	0.000 (0.000)^***^	0.010 (0.002)^***^
	Individual level variance–within	3.560/3.069	0.777/0.669	2.110/1.332
	Three level variance—between	1.353/0.825	0.099/0.059	1.524/0.226
	Pseudo *R*^2^ within	0.145	0.113	0.372
	Pseudo *R*^2^ between	0.365	0.131	0.844

*All analyses include the respective income and trust variables. Weighted N = 40,219*.

Analyses controlled for gender, age, age^2^, marital status, number in household, education, occupation, unemployed, being sick or disabled, mental health, and year. Personal income is measured as yearly household income, community income per thousand is measured aggregated mean of household income for country, region and social class; national income is measured as Ln GDP (PPP) per capita per thousand. Ranges of income: Personal income, −4.45–200; community income, −3–74; national income, 2.4–4.2; Ranges of trust: personal, 1–9; social, 0–30; political, 0–50. Ranges of satisfaction: Personal life satisfaction, 0–10; social satisfaction, 0.3–173.0; political satisfaction, 0–10. Levels in multilevel: individual, community (region and social class), and country. Random intercept. Restricted maximum likelihood. Residuals weighted for post-stratification weight. Significance:

* p < 0.05;

** p < 0.01;

*** *p < 0.001*.

Answers to **RQ2** (“Total holistic effect”) are shown in Table 3. Personal life satisfaction was significantly and positively associated with all three income variables and all three trust variables. Model 3 is thus an expression of the significant relationship between the macro, mezzo, and micro layers.

**Table 3:** Results [fixed effects (Beta (SE)sig) and pseudo *R*^2^ (explained variance) for four models of multilevel analysis of personal life satisfaction against personal trust and personal income (model 1); against personal parameters and additionally social trust and community income (model 2); the preceding parameters and additionally against political trust and national income (model 3); and finally the preceding parameters with the three interactions of all three income parameters, indicating a layer effect (model 4).

**Model**	**Null/demographic 0**	**Without trust**	**Individual 1**	**Community 2**	**Country 3**	**Interaction 4**
Personal income		0.007 (0.000)^***^	0.009 (0.000)^***^	0.006 (0.000)^***^	0.006 (0.000)^***^	0.021 (0.006)^**^
Personal trust			0.507 (0.012)^***^	0.475 (0.011)^***^	0.478 (0.011)^***^	0.477 (0.011)^***^
Community income		−0.002 (0.001)^NS^		0.010 (0.001)^***^	−0.003 (0.001)^**^	0.048 (0.010)^***^
Social trust				0.077 (0.002)^***^	0.055 (0.002)^***^	0.055 (0.002)^***^
National income		1.796 (0.055)^***^			1.293 (0.051)^***^	1.261 (0.066)^***^
Polit. trust					0.028 (0.001)^***^	0.028 (0.001)^***^
Int Ntlincome × Comincome						−0.012 (0.003)^***^
Int Ntlincome × Perinc						−0.003 (0.002)^NS^
Int comincome × perinc						−0.000 (0.000)^***^
Individual level variance—within	3.560/3.322	3.203	3.070	2.969	2.916	2.911
Three level variance—between	1.353/1.028	0.399	0.825	0.496	0.306	0.284
Pseudo *R*^2^ within	0/0.096	0.171	0.093	0.160	0.181	0.182
Pseudo *R*^2^ between	0/0.240	0.768	0.274	0.562	0.774	0.790

N = 44,833. Analyses controlled for gender, age, age^2^, marital status, number in household, unemployed, education, occupation, being sick or disabled, mental health, and year. Personal income is measured as yearly household income, community income per thousand is measured aggregated mean of household income for country, region and social class; national income is measured as Ln GDP (PPP) per capita per thousand. Ranges of income: Personal income, −4.45–200; community income, −3–74; national income, 2.4–4.2; Ranges of trust: personal, 1–9; social, 0–30; political, 0–50. Range of personal life satisfaction, 0–10. Levels in multilevel: individual, community, and country. Random intercept. Restricted maximum likelihood. Residuals weighted for post-stratification weight. Significance: NS = Not Significant;

* p < 0.05;

** p < 0.01;

*** *p < 0.001*.

Answers to **RQ3** (“Effect size of holistic approach”) are shown in Figure 2. Figure 2 shows these results by converting the betas to relative effects using the weighted mean values for all of Europe. On a variable by variable basis, national income (44%) had the largest relative effect on variation in personal life satisfaction, followed by personal trust (19%), community income (15%), while political trust (6%), social trust (9%), and personal income (7%) had the smallest effect.

Answers to **RQ4** (“The layers effects”) are also shown in Table 3 and Figure 2. The country layer contributed most to the variation in personal life satisfaction (51%), followed by the personal layer (26%) and finally the social layer (23%). The significant relationship between layers is further documented in model 4 (Table 3), by the significant negative interaction of the national income variable with the community income variable. This indicates that the effect of community income on personal life satisfaction is weaker in rich countries and stronger in poor countries. The interaction of the community and personal income variables was significant and negative. This indicates the effect of personal income on personal life satisfaction is weaker in rich communities than in poor communities. The interaction of the national with the personal income variables was not significant. This indicates no differences between either rich or poor countries in the relationship of personal income with personal life satisfaction. However, if the interaction of the national to the community income variable were removed (data not shown), the interaction between the national and personal income variables became significantly negative [−0,008 (0.001)^***^]. This indicates that the layer effect goes through the community layer. The pseudo *R*^2^ values were high, both on an individual level (18% of variation explained) and especially between the levels (79% of variation explained). However, the pseudo *R*^2^ values also indicate that the variables representing the sociodemographic parameters explain some of the variance within (10%) and especially between the levels (24%). The results of the full analysis (model 4), including confounders, is presented in Supplementary Material 3, Table 5.

Answers to **RQ5** (“The themes effects”) are also shown in Figure 2. Thematically, income had the greatest relative association with personal life satisfaction (66%), followed by trust (34%).

Answers to **RQ6** (“Variation by countries”) are shown in Table 4 which shows results of the multilevel analysis of model 4 in Table 3 sorted by countries grouped by their levels of trust. In Group 1 (High-trust levels) and Group 2 (Medium-trust levels) personal life satisfaction was not significantly associated with any of the three income variables. However, in Group 3 (Low-trust) all the three income variables were highly and significantly associated with personal life satisfaction. This indicates that income is an important factor in explaining variation in personal life satisfaction, but only in countries with low overall trust. The associations of the three trust parameters with personal life satisfaction were significant in all three groups, increasing in importance from high to low-trust for personal and political trust. This indicates that trust, be it personal or national, is of greater importance in countries with low overall trust. For social trust, however, this effect decreased from Groups 1 and 2 to Group 3, indicating the reverse. That is that as levels of overall trust in countries decreases, the importance of social trust also decreases. The interaction effects are an indication of the effects of the different layers. This indicates that within the group of countries with low overall trust, the relationships between personal and community income and personal life satisfaction are stronger in the wealthier countries than in the poorer countries (negative interaction between national and personal income). Similarly, the layer effect of community to personal income indicates that in the richer communities, especially within those countries in the low overall trust group, higher personal income is associated with higher personal life satisfaction than it is in poor communities (negative interaction between community income and personal income).

**Table 4:** Results three models [fixed effects (Beta (SE)sig) and pseudo *R*^2^ (explained variance)] for three models (based on country trust groups) of multilevel analysis of personal life satisfaction (at individual level) against personal, social and national income and against personal, social and political trust (model 4 in Table 3).

**Model**	**Low trust 3**	**Medium trust 2**	**High trust 1**
*N*	12,296	16,329	16,208
Personal income	0.104 (0.030)^***^	0.019 (0.018)^NS^	−0.006 (0.009)^NS^
Community income	0.133 (0.056)^*^	0.014 (0.039)^NS^	0.011 (0.016)^NS^
National income	1.824 (0.203)^***^	−0.739 (0.341)^NS^	−0.267 (0.249)^NS^
Personal trust	0.509 (0.026)^***^	0.482 (0.019)^***^	0.430 (0.015)^***^
Social trust	0.044 (0.004)^***^	0.062 (0.003)^***^	0.056 (0.003)^***^
Political trust	0.036 (0.002)^***^	0.028 (0.002)^***^	0.019 (0.001)^***^
Int National income × Community income	−0. 034 (0.017)^*^	0.003 (0.010)^NS^	−0.002 (0.004)^NS^
Int National income × Personal income	−0.026 (0.009)^**^	−0.001 (0.005)^NS^	0.004 (0.002)^NS^
Int Community income × Personal income	−0.000 (0.000)^***^	0.000 (0.000)^***^	−0.000 (0.000)^***^
Individual level variance—within	4.700/3.966	3.989/3.184	2.290/1.795
Three level variance—between	1.532/0.485	0.417/0.174	0.091/0.031
Pseudo *R*^2^ within	0.181	0.160	0.093
Pseudo *R*^2^ between	0.800	0.562	0.274

Analyses controlled for gender, age, age^2^, marital status, number in household, education, occupation, unemployment, being sick or disabled, mental health, and year. Personal income is measured as yearly household income, community income per thousand is measured as aggregated mean of household income for country, region, and social class; national income is measured as Ln GDP (PPP) per capita per thousand. Ranges of income: Personal income, −4.45–200; community income, −3–74; national income, 2.4–4.2; Ranges of trust: personal, 1–9; social, 0–30; political, 0–50. Range of personal life satisfaction, 0–10. Levels in multilevel: individual, community, and country. Random intercept. Restricted maximum likelihood. Residuals weighted for post-stratification weight. Significance: NS = Not Significant;

* p < 0.05;

** p < 0.01;

*** *p < 0.001*.

### The Buffer Hypothesis

Answers to **RQ7** (“The buffer hypothesis”) are shown in Tables 2, 5. Table 2 presents the results of the analyses within layers. The interaction term between personal trust and personal income was significantly negative. This indicates that there is a moderator effect of personal trust, such that with increased personal trust the relationship between personal income and personal life satisfaction becomes less intensive, or vice versa. The interaction term between social trust and community income was significant and positive. This indicates that social trust moderates the relationship between community income and social satisfaction such that as social trust levels increase, the strength of the negative association with low community income increases. Finally, at the country layer the interaction term between political trust and national income was significant and positive. This indicates that political trust is a significant moderator of the relationship between national income and political satisfaction. Consequently, as political trust increases, the relationship between political satisfaction and national income becomes intensified. Or the opposite, if political trust is decreases, the association between national income and political satisfaction becomes weaker.

**Table 5:** Results of moderation analysis (using Hayes PROCESS, which does not allow multilevel) for the moderator role of personal, social and political trust for X = personal income and Y = PLS, by country group as defined by overall trust and for the overall population.

**Country group**	**Moderator**		**Effect on income parameter**		
	**Trust**	**Levels of moderator**	***N***	**Effect on X(SE)Sig**	***R*^**2**^/Interaction sig**
Total	Personal	Low	55,253	0.023 (0.001)^***^	0.228/−0.003^***^
		Medium		0.020 (0.000)^***^	
		High		0.016 (0.001)^***^	
	Social	Low	54,891	0.022 (0.001)^***^	0.265/−0.001^***^
		Medium		0.016 (0.000)^***^	
		High		0.010 (0.000)^***^	
	Political	Low	52,909	0.023 (0.001)^***^	0.271/−0.0007^***^
		Medium		0.014 (0.000)^***^	
		High		0.007 (0.000)^***^	
High	Personal	Low	18,523	0.006 (0.001)^***^	0.186/−0.001^***^
		Medium		0.004 (0.000)^***^	
		High		0.004 (0.000)^***^	
	Social	Low	18,485	0.005 (0.001)^***^	0.186/−0.0003^***^
		Medium		0.004 (0.000)^***^	
		High		0.003 (0.000)^***^	
	Political	Low	17,992	0.005 (0.001)^***^	0.168/−0.0002^***^
		Medium		0.003 (0.000)^***^	
		High		0.003 (0.000)^***^	
Medium	Personal	Low	19,447	0.015 (0.001)^***^	0.184/−0.005^***^
		Medium		0.010 (0.001)^***^	
		High		0.010 (0.001)^***^	
	Social	Low	19,382	0.014 (0.001)^***^	0.195/−0.0009^***^
		Medium		0.010 (0.001)^***^	
		High		0.005 (0.001)^***^	
	Political	Low	18,843	0.013 (0.001)^***^	0.186/−0.0003^***^
		Medium		0.009 (0.001)^***^	
		High		0.006 (0.001)^***^	
Low	Personal	Low	17,283	0.045 (0.003)^***^	0.194/−0.006^*^
		Medium		0.040 (0.002)^***^	
		High		0.035 (0.003)^***^	
	Social	Low	17,024	0.050 (0.002)^***^	0.192/−0.001^***^
		Medium		0.040 (0.002)^***^	
		High		0.032 (0.002)^***^	
	Political	Low	16,074	0.055 (0.003)^***^	0.215/−0.0014^***^
		Medium		0.039 (0.002)^***^	
		High		0.023 (0.002)^***^	

Analyses controlled for gender, age, age^2^, marital status, number in household, unemployed, education, occupation, being sick or disabled, mental health, and year. Personal income is measured as yearly household income. Range of personal income −4.45 to 200. Ranges of trust: personal, 1–9; social, 0–30; political, 0–50. Range of personal life satisfaction, 0–10. Levels in multilevel: individual, community, and country. Random intercept. Restricted maximum likelihood. Residuals weighted for post-stratification weight. Significance:

* p < 0.05;

** p < 0.01;

*** *p < 0.001*.

Table 5 presents the results of moderation analyses for the X–Y relationship personal income—personal life satisfaction, total and within each country group. It indicates that, for the entire population, all three forms for trust, personal, social, and national, are significant negative moderators. Although all three forms of trust are also significant negative moderators within each country group, the effect levels of the medium groups is approximately double the effect of the high trust group, whereas the effect size of the low trust group is a little <10 times the effect. Since the interaction is negative, the level of effect decreases with increasing levels of trust.

## Discussion and Conclusion

Good public health and a high level of well-being and satisfaction with one's personal life are associated with personal, community and national income, as well as personal, social and political trust. However, how these measures relate to each other within and between countries and within and across different socio-structural layers of society, has been largely unknown. To answer these questions, we launched a three-layer nested, socio-structural model, similar to the Dahlgren and Whitehead's “rainbow model” (19).

The original “rainbow model” was launched primarily as a policy tool to handle social inequality of factors threatening, promoting and protecting health. The model stressed the importance of three socio-structural layers of society, the major structural environment, the material and social conditions, and the individual. Furthermore, it assumed that both direct and indirect effects of the layers were involved. In that respect it allowed policy makers a better perspective on the ramifications of suggested policy reforms.

Our model is constructed for research purposes. Similar to “the rainbow model,” it consists of three layers (individual, community, and country). Each layer contains a corresponding measure of income (personal, community, and national), trust (personal, social and political), and satisfaction (personal, social, and national). Combined with multilevel data-analytic techniques we used this model to analyze data from the European Social Survey in 19 countries divided into three groups according to their levels of social and political trust.

A particular interest of this study was to test what we have called “the buffer hypothesis,” that personal, social and political trust acts as a buffer against effects of personal income on satisfaction with one's personal life, and hence dampen effects of social inequality on the sense of well-being.

The buffer hypothesis. Our results give strong support to the buffer hypothesis. Both across European countries and within all three groups of countries, whether they are characterized by low, medium or high levels of social and political trust, the results are consistent with the buffer hypothesis. All three forms of trust dampen the effects of personal income on satisfaction with one's personal life. The more you trust yourself, your neighbors or the political and regulatory authorities in your country, the less important is personal income for how satisfied you are with your personal life. Or the other way around, the less you trust yourself, your neighbors or how the country is run, the more important is your personal income for how satisfied you are with your personal life. That is, trust, whether it is personal, social or political, compensates for the effect of low personal income on your personal satisfaction with life, and hence reduces differences in sense of well-being caused by economic inequality, both between individuals, between local communities, and between countries.

At the community and national layer, however, we observed no such buffer effect. There we observed that high trust in the local social environment or the country's political or regulatory authorities occurred primarily in areas and countries where income was high. In these areas and countries, the relationships between community income and social satisfaction and between national income and political satisfaction were associated with social and political trust, respectively. In fact, all or nearly all of the effects of income on satisfaction either with the neighborhood or with how the country was run, were due to social and political trust, respectively. Although some literature exists indicating a moderator role of social trust on well-being (2–5), to our knowledge there are really no studies to compare these findings with.

Variation by country. Furthermore, we found that satisfaction with one's personal life is also directly associated with personal, social and political trust, irrespective of the level of social and political trust in the country. However, such trust is far more important to personal life satisfaction in countries with low levels of social and political trust as compared to countries with medium or high levels of such trust. The less social and political trust there is in a country, the more important is such trust for satisfaction with one's personal life, both directly, and indirectly by dampening the effect of income.

When all measures of income, trust, and satisfaction were controlled against each other, the results show that in countries with high- or medium levels of social and political trust, personal satisfaction with life is associated neither with national, community nor personal income. In countries with low levels of trust, however, personal satisfaction with life is strongly associated with all three measures of income.

The dampening effect of trust in countries with a medium level of trust, such as France, Germany, Spain, Ireland, and UK, is approximately the double of the effect of that in countries with a high level of trust, such as the Nordic countries. Even more, the dampening effect of overall trust in countries with a low level of trust, such as Bulgaria, Poland, Slovakia, Slovenia, Portugal, and Cyprus, is nearly 10 times as big as in the countries with high level of trust. Altogether, these findings too are consistent with “the buffer hypothesis.”

A substantially higher significance of political trust to well-being in transition countries as compared to economically more developed countries in Europe, as well as a reduced association in the Nordic countries have been reported earlier (29, 30). One study reported that the relationship between personal life satisfaction and perceived quality of society was weaker in wealthier countries while the opposite was true in poorer countries (31). This could possibly be another indication of a relationship between political trust and satisfaction with one's personal life, which may indicate that the marginal effect of income is less in the high trust countries, where income is higher (29).

Total holistic effect. Another important finding of this study is that personal life satisfaction is positively linked to all three measures of income (personal, community, and national) as well as all three measures of trust (personal, social and political) when all measures of income, trust, and satisfaction are controlled against each other across countries. In our model, the strongest genuine effect on personal life satisfaction comes from national income (44%), then personal trust (19%), then community income (15%). While, may be surprising to some, political trust (6%), social trust (9%), and personal income (7%) have only minor effects.

As mentioned in the introduction, positive separate associations between several of the themes in this study, have been documented before. However, to our knowledge no study has compared the unique, relative contributions of these measures to personal life satisfaction in a multilevel model where all variables are controlled for each other.

Layer effects. When comparing effects of socio-structural layers, we found that there is a significant and substantial effect of the outer layers on the individual. Or the other way around, the individual layer was associated with the community layer which was associated with the country layer.

However, when we looked at which socio-structural layer (individual, community, and country) of the society that contributed the most to satisfaction with one's personal life, we found that more than half of the contribution comes from the country level, and that the social and personal level contribute with about a quarter each. This indicates that in the long run, what happens at the country level, may be far more important to one's personal life satisfaction than what happens at the community or individual level.

At the country level, the significance of national income, political trust and political satisfaction to personal life satisfaction, has been documented in many countries. Good government contributes to well-being by providing high quality technical delivery of goods, welfare, and democratic values. The relationship between good government and well-being involves both direct effects on individual happiness and indirect effects through educational and other reforms that help individuals realize factors important to well-being (25, 32), as well as civic nationalism (33, 34).

At the community level, it has been observed that political trust and political satisfaction may be more important than social trust in predicting personal life satisfaction (35). Several studies have also shown the significance of the community layer on well-being, emphasizing the importance of size of the geographic groups as for example the neighborhood and larger geographical units (5, 8, 13).

However, no one has ever demonstrated such layer effects within such a comprehensive multilevel context as in the current study.

Thematic effects. What then is most important, to satisfaction with one's personal life; is it income or trust? Our study shows that it's the money. In our model, two thirds of the effect come from income (personal, community, and national), while one third comes from trust (personal, social and political).

In general, our findings on the significance of personal, community, and national income to well-being are in concert with a large number of previous studies (5, 6, 8–11, 13, 14, 18, 20, 33, 36–38). Personal income, also called absolute income, seems to influence well-being in a positive direction (6, 10). Much of this discussion concerns the association of income to material consumption.

In the literature, the effect of community income may vary considerably according to the size of the “community,” i.e., whether it is a state or province or a neighborhood. At the larger geographical level, e.g., provinces in Canada, a negative association with well-being has been found. However, at the smaller level, e.g., neighborhood, the majority of studies show positive associations between community income and well-being (5, 7, 8, 12, 39). At the larger level, community income is believed to reflect availability of public goods. At the smaller level, community income most likely reflects private consumption (5, 8, 9, 11, 14, 15, 39). As the size of the community level becomes smaller, also other factors such as trust and security seem to enter the picture (8).

Well-being also varies between countries. Differences between countries in national income, e.g., Gross Domestic Product (GDP), may explain some of this variation. One study reported a strong, positive relationship between national income and subjective well-being (33), but this has been contested by the Easterlin paradox (18, 40, 41). Additionally, it has been shown that national income explains more of the variation in well-being than does personal income (38).

Our study extends current knowledge by accounting for both personal, community, and national income; and personal, social and political trust in one and the same multilevel model. Even within this comprehensive model, among the income measures, it is the country's national income that counts by far the most for how satisfied one is with one's personal life. Personal income is of only minor significance.

Mechanisms. What then are the mechanisms behind these findings on trust? Being a cross-sectional study, this study cannot say anything about that. But, should we speculate based on both ours and previous findings, we would put our money on the plain thesis that trust simply makes life simpler, easier, more pleasant and friendly, brings people closer together, reduces bureaucracy, and facilitates the economy. This way, personal, social and political trust serve as a kind of lubricant for the individual, the community, and the society at large (1, 42).

In public health, social trust is strongly associated with individual happiness, altruistic attitudes, simpler collaboration between people, sense of control of one's life, and better chances in life (43–45). Economically, social trust is associated with less formalities, conflicts, legal processes, lower transaction costs in commerce and favorable conditions for investment (46–48). Politically, social and political trust seems to promote political engagement and democratic development, and to reduce criminality (49, 50). The other way around, it is also probable that economic growth generates social trust, which in turn generates further growth and the other way around (51).

If we should dare to continue speculating further and go as far as to assume that our findings reflect underlying causal mechanisms, these findings would imply that politicians, professionals and regulators who want a satisfied electorate or population, should invest in enhancing the level of trust, both personal, social and political, in the population they are serving. This is partly because there is a direct relationship between trust and satisfaction at all socio-structural layers of society, and partly because trust in its different forms plays a strong and consistent role as a buffer against the effects of personal income on our personal life satisfaction, and thus dampens the effects of economic inequality on the inhabitants' sense of well-being, and ultimately, their public mental health.

In conclusion, the results of the model presented in this study may provide the authorities with a framework for policies aimed at improving the general well-being of their population, necessary for the healthy functioning of society. Thus, we join with the Nordic Council of Ministers' “manual” on how states can act to increase social and political trust over the long term (1): “Act with openness and transparency, manage tax revenues with respect, and tackle all signs of corruption, however negligible they seem. Create a general welfare state that prevents underclasses developing in society. Support associations, not least financially. It is generally favorable if the state can have an open attitude to associations. Raise the level of education in the population. Because of the importance of retaining relative economic homogeneity in the population, it is probably particularly important to focus on those with, or at risk of, low and/or incomplete education. Counteract unemployment, particularly long-term unemployment. This particularly implies efficient integration of refugees and immigrants in the labor market”

## Strengths and Limitations

One major strength of this study is that we used a combination of multilevel analysis and a three levels socio-structural model. This way, we could holistically assess how each layer-specific theme of income, trust and satisfaction is uniquely and independently associated with personal life satisfaction, controlling for all the others.

Another major strength of this study is the large sample size obtained by ESS and their use of methodological standards at all stages in the process. This makes the data ideal for comparative and cross-national analyses. The ESS team is working continuously to ensure high validity and reliability of the questionnaire and data collected. The use of strict randomized probability sampling provides a representative sample of the population, and the questionnaire used is well-tested and translated according to ESS protocols.

A third strength is that it includes comparable data across 19 European countries. This made it possible both to study the relative effect of the national layer as such and to come closer to theorizing about Europe as a whole. However, although more countries were included than in any previous similar study, the after all limited number of countries limits generalization to all Europe. Inclusion of data from other countries might have given somewhat different results, but were not available for the period examined in this paper.

This study has several other limitations too. The cross-sectional nature of the survey limits the possibility to draw causal conclusions from the findings. Consequently, we have only been able to speculate about the mechanisms that make trust such a powerful moderator of the relationship between income and satisfaction.

We used cumulative data from 2006 to 2012. This reflects particular periods in time. Joining of these time periods have been done under the assumption that the political climate and overall social discourse in these 2 years do not differ significantly.

Also because of the cross-sectional design, we were only able to examine short-term effects. In the long-term, the relationships we have revealed may be changed (52).

Furthermore, data were collected through self-report, and thus response bias might be present. However, several of the measures, such as those on trust and satisfaction, are truly subjective measures and can hardly be measured validly by other methods than by asking people.

The items used to measure satisfaction and trust at all three levels could have been more consistent across levels.

The ESS includes no standard measure on trust in oneself. However, although not 100% perfect, the concepts of self-confidence and self-esteem are logically very close. We therefore used self-esteem as a proxy for trust in oneself.

The data needed to determine measures on the community level were only available for two rounds (3 and 6), resulting in a lower samples size. At the community level, information about the neighborhood was not available. We therefore used regional level within countries to represent the local community. However, it has been shown that larger regional units represent more satisfactorily the community effect than estimates at the neighborhood level (53). To address this problem, we combined information on the regions within the country that the individuals lived in together with the social class. Although this was not ideal, it was considered an acceptable approximation.

Unfortunately, the income variable was changed in 2008 from 12 identical categories to 10 specific for each country. However, we controlled for this by imputing a personal income for each respondent using nation-specific information on the distribution by gender, age, and education.

## Data Availability Statement

Publicly available datasets were analyzed in this study. This data can be found at: European Social Survey https://www.europeansocialsurvey.org/data/.

## Ethics Statement

The data are available without restrictions, for not-for-profit purposes. In accordance with the ESS ERIC Statutes (Article 23.3), the ESS ERIC subscribes to the Declaration on Professional Ethics of the International Statistical Institute. The Research Ethics Committee reviews applications for studies for which the ESS ERIC is directly responsible, that is, which it directly contracts. Written informed consent to participate in this study was provided by the participants' legal guardian/next of kin.

## Author Contributions

JC-A and AH contributed to the conception, design of the work, contributed to further drafts, revisions of the article, and contributed to the final approval of the version to be published. JC-A was responsible for data analysis and interpretation as well as writing the first draft of the article. Both authors contributed to the article and approved the submitted version.

## Conflict of Interest

The authors declare that the research was conducted in the absence of any commercial or financial relationships that could be construed as a potential conflict of interest.
