# Supplementary material for: Political Trust Influences the Relationship Between Income and Life Satisfaction in Europe: Differential Associations With Trust at National, Community, and Individual Level
Source: Front Public Health. 2021 Mar 15;9:629118. doi: 10.3389/fpubh.2021.629118 (PMC8005631; doi:10.3389/fpubh.2021.629118)
Supplement: Supplementary file 1 [file Data_Sheet_1.docx]

**Supplementary material 1 – Literature review**

This appendix provides a total, detailed literature review of the subject matter.

Although researchers have debated whether personal trust has any influence on important life outcomes [1-4], a growing body of evidence supports the notion that *personal trust* in terms of self-esteem does have consequences for people’s lives. One summary shows that there is a close relationship between high self-esteem and happiness [5-9], a relationship that studies, including longitudinal studies, indicate are in the direction of self-esteem to happiness [1, 10-13]. In particular, prospective studies suggest that self-esteem influences people’s success and well-being in the domains of social relationships [14-17], mental health [18-23], school and education [7, 8], work [5-8], and physical health [6, 7, 24]. For other reviews, see [24-27]. Altogether, these studies allow for relatively strong conclusions because many of the studies used large and representative samples, controlled for prior levels of the outcomes, and controlled for confounding factors such as gender, socioeconomic status, intelligence, and life events [28].

*Social trust,* also called interpersonal trust, concerns the expectation that people do not have dishonest intentions. Social trust is strongly associated with well-being in terms of happiness or life satisfaction (LS). When examining national differences in well-being, social trust has been shown to constitute a powerful explanatory factor [29-32]. People simply feel good about living in a society where people trust each other [33]. One aspect of this may be that social trust simplifies collaboration and promotes altruistic preferences in the population [34]. In general, people with high levels of social trust are more inclined to perceive that they have greater control of their lives and have better life chances [35, 36].

Economically, individuals, local communities and countries benefit from social trust in many ways [37]. Social interaction and economic transactions are less characterized by formalities, conflicts, legal processes, needs to verify other people’s actions [38], needs for control in working life [39], and transaction costs connected to ensuring compliance with agreements which ultimately takes time from productive work [40, 41]. Another economic benefit is that high levels of trust seems to help create favorable conditions for investment [38]. This way, social trust serves as a kind of lubricant for the economy [41-43]. One review of empirical studies calculated that an increase of ten percentage points in social trust can be expected to increase economic growth by half a percentage point [44].

Extending measured results of social trust from the individual to the societal level, enables comparisons of the social capital between different societal and geographical areas. Differences in social trust can to a large degree explain national differences in well-being [29-31]. Association between social trust and personal and national income (Gross Domestic Product, GDP) has been observed. This association is probably bidirectional [45]. An association between social trust and social class has also been observed [46]. The effect of social trust on well-being has been shown to exceed that of GDP over the long term. The effect of GDP, which was prominent over the short-term, was considerably reduced in favor of social trust over the long-term [47].

Well-being in terms of both happiness [48] and LS [49] seems to be associated with trust in a country’s institutions, called *political trust* or sometimes national trust [32, 48, 50]. One study found political trust and satisfaction with how the country is run, called *political satisfaction* or sometimes national satisfaction, to be stronger predictors of well-being than were trust in one’s social environment [49]. However, these findings were not confirmed in the USA [51]. The actual performance of the institution seems to influence the evaluation of political trust [31, 48, 52, 53]. Unfortunately, less is known about the role of political trust on political satisfaction.

Another factor that seems to affect well-being is income. *Personal income,* also called absolute income, seems to influence well-being in a positive direction [54, 55]. Much of this discussion concerns the association of income to material consumption. As regards, *community income,* a negative association with well-being has been found. But that is at the largest geographical level, e.g. provinces in Canada [56-60]. At the smallest level, e.g. neighborhood, the majority of studies observed positive associations between community income and well-being. This difference between the two sizes of communities is believed to reflect availability of public goods at the larger level and consumption at the smaller level [56, 58, 60-64].

However, the concept of average community income at the local level, is rather complex. It may reflect the overall wealth of the nation or group consumption as reflected in finer stores, homes, cars etc. in the richer communities, or crime, social and mental health problems etc. in the poorer communities [60]. As the size of the local level becomes smaller, also other factors such as trust and security seem to enter the picture [60]. At the local level, the findings sometimes reflect relative income rather than community income [60].

Well-being also varies between countries. For example, the Nordic countries have consistently been shown to have the highest levels of well-being in the world. Whereas countries in Eastern Europe score low in a European context [65-67]. Differences between countries in *national income*, e.g. Gross Domestic Product (GDP), may explain some of this variation. One study reported a strong, positive relationship between national income and subjective well-being [68]. Another explanatory factor might be differences in income inequality [69].

A central thesis in the discussion about the effect of income on well-being has been the Easterlin paradox [70-72]. Easterlin claimed that: “At a point in time happiness varies directly with income, but over time happiness does not increase when a country’s income increases”. Contesters to the paradox claim that the well-known relationship between well-being and GDP is linear with no upper satiation point [70-74]. Easterlin has rebutted this by claiming that these changes represent short-term changes in GDP, not long-term changes. Additionally, it has been shown that national income explains more of the variation in well-being than does personal income [75].

With few exceptions [68, 76], most of these studies address individual well-being, e.g. in terms of *personal LS,* not how satisfied individuals are with the social environment of their local community, e.g. closeness, respectfulness and helpfulness, called *social satisfaction,* or how satisfied they are with how their country is run. However, one study found that personal income is associated with social satisfaction [76]. Another study found that also community income is associated with social satisfaction [76]. Furthermore, one study found a strong positive association between national income and national satisfaction [53, 68].

However, although discussed extensively [71-74], overall, there is still uncertainty about which, how, and to what extent economic factors influence well-being [55, 71, 77, 78]. In particular, knowledge is lacking about the relative contribution of different types of income, e.g. personal, community or national, to different *facets of well-being*, including personal, social and political satisfaction. Is it personal income, community income or national income that matters the most, and which one of these matters the most to which facet of well-being [54, 55, 71, 77, 78]? Neither do we know much about how personal income and national income relate. Indications in the literature are mixed with both positive interaction [75] and negative [79]. Also, unfortunately, all these studies have been conducted in single countries and therefore do not reflect the association of the country level of income.

Trust is frequently used as an indicator of a society’s social capital [31, 80]. A moderating role of social capital has been suggested in different contexts. Moderation occurs when the direction and/or strength of a relationship between two variables depends on a third variable (interaction). A negative moderation indicates that the relationship between the dependent and independent variable, for example the slope, is weakened or flattened. A positive moderation means that it is strengthened, or the slope becomes steeper. For instance, one study found a moderating role of social capital on the relationship between national income (GDP) and well-being [81]. A moderating role of social capital in terms of resilience when exposed to different forms of adversity, has been reported [31, 82, 83]. Also, of note, one study reported that the relationship between well-being and perceived quality of society was weaker in countries with wealthy economies while the opposite was true in countries with deprivation [65]. However, precise studies on the moderating role of trust are scarce.

Altogether, with more or less certainty, it may seem that well-being, social satisfaction and political satisfaction are associated with trust and income in one way or other. Sometimes the effects of trust even seem to exceed the effects of income. We may therefore assume that in a negative moderation, when the level of trust is high, the association between income and well-being, social and political satisfaction will be weaker. And vice versa, when the level of trust is low, the association between income and well-being, social and political satisfaction will be stronger. Visualize: Following a fall in income, well-being will be less impaired when levels of trust are high than when levels of trust are low. In more technical terms, we hypothesize that trust, either personal, social or political, acts as a moderator of the relationships between income and satisfaction.

Since there are indications of association between well-being and both personal, community and national income, we may also expect that different domains of trust (e.g. personal, social, political), have separate relative associations at different structural levels of a society (e.g. individual, community, country) and to have separate moderating effect on personal, social and political satisfaction, respectively.

Furthermore, we suggest that part of the uncertainty about how income influences well-being [84-87] may be related to lack of accounting for the relative associations of factors at different socio-structural layers of the society (e.g. individual, community, country) [79, 88]. Since both personal, community and national income have been found to be associated with well-being, these economical determinants should be put together in one holistic model to be able to assess their relative contribution to a personal life satisfaction, social and political satisfaction. Although empirical support is currently weak, a corresponding way of reasoning could be applied to the association between trust and well-being.

Finally, since the relationship between well-being and perceived quality of society seems to be weaker in countries with wealthy economies while the opposite seems to be true in countries with deprivation [65], we assume that the relationship between income and well-being will vary between countries according to their levels of trust.

1. Baumeister RF, Campbell JD, Krueger JI, Vohs KD. Does high self-esteem cause better performance, interpersonal success, happiness, or healthier lifestyles? Psychological science in the public interest. 2003;4(1):1-44.

2. Krueger JI, Vohs KD, Baumeister RF. Is the allure of self-esteem a mirage after all? American psychologist. 2008;63(1):64-5. doi: 10.1037/0003.066X.63.1.64.

3. Swann Jr WB, Chang-Schneider C, Larsen McClarty K. Do people's self-views matter? Self-concept and self-esteem in everyday life. American psychologist. 2007;62(2):84.

4. Swann Jr WB, Chang-Schneider C, McClarty KL. Yes, cavalier attitudes can have pernicious consequences. American psychologist. 2008;63(1):65-6.

5. Kuster F, Orth U, Meier LL. High self-esteem prospectively predicts better work conditions and outcomes. Social Psychological and Personality Science. 2013;4(6):668-75.

6. Orth U, Robins RW, Widaman KF. Life-span development of self-esteem and its effects on important life outcomes. Journal of personality and social psychology. 2012;102(6):1271.

7. Trzesniewski KH, Donnellan MB, Moffitt TE, Robins RW, Poulton R, Caspi A. Low self-esteem during adolescence predicts poor health, criminal behavior, and limited economic prospects during adulthood. Developmental psychology. 2006;42(2):381.

8. von Soest T, Wichstrøm L, Kvalem IL. The development of global and domain-specific self-esteem from age 13 to 31. Journal of personality and social psychology. 2016;110(4):592.

9. Orth U, Erol RY, Luciano EC. Development of self-esteem from age 4 to 94 years: A meta-analysis of longitudinal studies. Psychological bulletin. 2018;144(10):1045.

10. Lyubomirsky S, Tkach C, DiMatteo MR. What are the differences between happiness and self-esteem. Social Indicators Research. 2006;78(3):363-404.

11. Margolis S, Lyubomirsky S. Cognitive outlooks and well-being. Handbook of Well-Being Noba Scholar Handbook series: Subjective well-being Salt Lake City, UT: DEF publishers DOI: nobascholar com. 2018.

12. Clench-Aas J, Holte A. The financial crisis in Europe: impact on satisfaction with life. Scandinavian journal of public health. 2017;45(18_suppl):30-40.

13. Moza D, Maricuțoiu L, Gavreliuc A. Cross-lagged relationships between self-esteem, self-construal, and happiness in a three-wave longitudinal study. Journal of Individual Differences. 2019;40(3):177.

14. Johnson MD, Galambos NL. Paths to intimate relationship quality from parent–adolescent relations and mental health. Journal of Marriage and Family. 2014;76(1):145-60.

15. Marshall SL, Parker PD, Ciarrochi J, Heaven PC. Is self‐esteem a cause or consequence of social support? A 4‐year longitudinal study. Child Development. 2014;85(3):1275-91.

16. Mund M, Finn C, Hagemeyer B, Zimmermann J, Neyer FJ. The dynamics of self‐esteem in partner relationships. European Journal of Personality. 2015;29(2):235-49.

17. Orth U, Robins RW, Widaman KF, Conger RD. Is low self-esteem a risk factor for depression? Findings from a longitudinal study of Mexican-origin youth. Developmental psychology. 2014;50(2):622.

18. Orth U, Robins RW, Meier LL, Conger RD. Refining the vulnerability model of low self-esteem and depression: Disentangling the effects of genuine self-esteem and narcissism. Journal of personality and social psychology. 2016;110(1):133.

19. Orth U, Robins RW, Roberts BW. Low self-esteem prospectively predicts depression in adolescence and young adulthood. Journal of personality and social psychology. 2008;95(3):695.

20. Sowislo JF, Orth U. Does low self-esteem predict depression and anxiety? A meta-analysis of longitudinal studies. Psychological bulletin. 2013;139(1):213.

21. Sowislo JF, Orth U, Meier LL. What constitutes vulnerable self-esteem? Comparing the prospective effects of low, unstable, and contingent self-esteem on depressive symptoms. Journal of Abnormal Psychology. 2014;123(4):737.

22. Steiger AE, Allemand M, Robins RW, Fend HA. Low and decreasing self-esteem during adolescence predict adult depression two decades later. Journal of personality and social psychology. 2014;106(2):325.

23. Wouters S, Duriez B, Luyckx K, Klimstra T, Colpin H, Soenens B, et al. Depressive symptoms in university freshmen: Longitudinal relations with contingent self-esteem and level of self-esteem. Journal of Research in Personality. 2013;47(4):356-63.

24. Orth U, Robins RW. Understanding the link between low self-esteem and depression. Current directions in psychological science. 2013;22(6):455-60.

25. Donnellan MB, Trzesniewski KH, Robins RW. Self-Esteem: Enduring issues and controversies. In: T. Chamorro-Premuzic SvS, A. Furnham editor. The Wiley-Blackwell handbook of individual differences. Chichester, UK: Wiley-Blackwell; 2011. p. 718-46.

26. Orth U. The family environment in early childhood has a long-term effect on self-esteem: A longitudinal study from birth to age 27 years. Journal of personality and social psychology. 2018;114(4):637.

27. Orth U, Robins RW. The development of self-esteem. Current Directions in Psychological Science. 2014;23(5):381-7.

28. Orth U, Robins RW. Development of self-esteem across the lifespan. In: D. P. McAdams RLS, J. L. Tackett, editor. Handbook of personality development. NY, USA: The Guilford Press; 2019. p. 328-44.

29. Bjørnskov C. The happy few: Cross–country evidence on social capital and life satisfaction. Kyklos. 2003;56(1):3-16.

30. Helliwell JF. How's life? Combining individual and national variables to explain subjective well-being. Economic modelling. 2003;20(2):331-60.

31. Helliwell JF, Huang H, Wang S. New evidence on trust and well-being. National Bureau of Economic Research, 2016.

32. Glatz C, Eder A. Patterns of Trust and Subjective Well-Being Across Europe: New Insights from Repeated Cross-Sectional Analyses Based on the European Social Survey 2002–2016. Social Indicators Research. 2019:1-23.

33. Rodríguez-Pose A. Social Capital and Individual Happiness in Europe. Bruges European Economic Research (BEER) Papers 25/December 2012. 2012.

34. Putnam RD, Leonardi R, Nanetti RY. Making democracy work: Civic traditions in modern Italy: Princeton university press; 1994.

35. Leung A, Kier C, Fung T, Fung L, Sproule R. Searching for happiness: The importance of social capital. The exploration of happiness: Springer; 2013. p. 247-67.

36. Rothstein B. Corruption and social trust: Why the fish rots from the head down. social research. 2013;80(4):1009-32.

37. Tabellini G. Culture and institutions: economic development in the regions of Europe. Journal of the European Economic association. 2010;8(4):677-716.

38. Zak PJ, Knack S. Trust and growth. The economic journal. 2001;111(470):295-321.

39. Ilsøe A. Between trust and control: company‐level bargaining on flexible working hours in the Danish and German metal industries. Industrial Relations Journal. 2010;41(1):34-51.

40. De Groot HL, Linders GJ, Rietveld P, Subramanian U. The institutional determinants of bilateral trade patterns. Kyklos. 2004;57(1):103-23.

41. Fukuyama F. Trust: The social virtues and the creation of prosperity: Free press New York; 1995.

42. Bjørnskov C. The Determinants of Trust (March 2005). Stockholm, SE: The Ratio Institute, 2006.

43. Blanchard O, Jaumott F, Loungani P. Unemployment, labour-market flexibility and IMF advice: Moving beyond mantras'. VoxEU (18 October) Available on the Internet: http://voxeu org/article/unemployment-labour-market-flexibility-and-imfadvice. 2013.

44. Bjørnskov C. Economic growth. In: Svendsen GT, Svendsen GLH, editors. Handbook of Social Capital. London: Edward Elgar; 2009. p. 3431-13.

45. Algan Y, Cahuc P. Trust, growth, and well-being: New evidence and policy implications. Handbook of economic growth. 2: Elsevier; 2014. p. 49-120.

46. Navarro-Carrillo G, Valor-Segura I, Moya M. Do you trust strangers, close acquaintances, and members of your ingroup? Differences in trust based on social class in Spain. Social Indicators Research. 2018;135(2):585-97.

47. Bartolini S, Sarracino F. Happy for how long? How social capital and economic growth relate to happiness over time. Ecological economics. 2014;108:242-56.

48. Hudson J. Institutional trust and subjective well‐being across the EU. Kyklos. 2006;59(1):43-62.

49. Mota GL, Pereira PT. Happiness, economic well-being, social capital and the quality of institutions. " Working Papers Department of Economics 2008/40, ISEG - Lisbon School of Economics and Management, Department of Economics, Universidade de Lisboa. 2008.

50. Khodyakov D. Trust as a process: A three-dimensional. Sociology. 2007;41(1):115-32.

51. Bartolini S, Bilancini E, Pugno M. Did the decline in social connections depress Americans’ happiness? Social indicators research. 2013;110(3):1033-59.

52. Helliwell JF, Huang H, Grover S, Wang S. Good governance and national well-being. OECD Working Papers on Public Governance, No 25, OECD Publishing, Paris,. 2014. doi: https://doi.org/10.1787/5jxv9f651hvj-en.

53. Helliwell JF, Huang H, Grover S, Wang S. Empirical linkages between good governance and national well-being. Journal of Comparative Economics. 2018;46(4):1332-46.

54. Biswas-Diener R. Material wealth and subjective well-being. The science of subjective well-being. 2008:307-22.

55. Diener E, Lucas RE, Oishi S. Advances and open questions in the science of subjective well-being. Collabra: Psychology. 2018;4(1).

56. Barrington-Leigh CP, Helliwell JF. Empathy and emulation: Life satisfaction and the urban geography of comparison groups. National Bureau of Economic Research, 2008.

57. Graf E, Tillé Y. Imputation of income data with generalized calibration procedure and GB2 law: illustration with SILC data. Proceedings 59th ISI World Statistics Congress, 25-30 August 2013, Hong Kong (Session CPS203). 2013:5509-14.

58. Kingdon GG, Knight J. Community, comparisons and subjective well-being in a divided society. Journal of Economic Behavior & Organization. 2007;64(1):69-90.

59. Blanchflower DG, Oswald AJ. Well-being over time in Britain and the USA. Journal of public economics. 2004;88(7-8):1359-86.

60. Brodeur A, Flèche S. Neighbors' income, public goods, and well‐being. Review of Income and Wealth. 2018;65(2):217-38.

61. Clark AE, Westergård-Nielsen N, Kristensen N. Economic satisfaction and income rank in small neighbourhoods. Journal of the European Economic Association. 2009;7(2-3):519-27.

62. Dittmann J, Goebel J. Your house, your car, your education: The socioeconomic situation of the neighborhood and its impact on life satisfaction in Germany. Social Indicators Research. 2010;96(3):497-513.

63. Knies G, Burgess S, Propper C. Keeping up with the schmidt’s: An empirical test of relative deprivation theory in the neighbourhood context. Schmollers Jahrbuch: Journal of Applied Social Science Studies. 2008;128(1):75-108.

64. Ma J, Dong G, Chen Y, Zhang W. Does satisfactory neighbourhood environment lead to a satisfying life? An investigation of the association between neighbourhood environment and life satisfaction in Beijing. Cities. 2018;74:229-39.

65. Böhnke P. Does society matter? Life satisfaction in the enlarged Europe. Social Indicators Research. 2008;87(2):189-210.

66. Michaelson J, Abdallah S, Steuer N, Thompson S, Marks N, Aked J, et al. National Accounts of Well-being: bringing real wealth onto the balance sheet. London: The New Economics Foundation, 2009.

67. Sachs JD, Layard R, Helliwell JF. World Happiness Report 2018. 2018.

68. Morrison M, Tay L, Diener E. Subjective well-being and national satisfaction: Findings from a worldwide survey. Psychological science. 2011;22(2):166-71.

69. Graafland J, Compen B. Economic freedom and life satisfaction: Mediation by income per capita and generalized trust. Journal of Happiness Studies. 2015;16(3):789-810.

70. Easterlin RA. Does economic growth improve the human lot? Some empirical evidence. Nations and households in economic growth. 1974;89:89-125.

71. Easterlin RA. Will raising the incomes of all increase the happiness of all? Journal of Economic Behavior & Organization. 1995;27(1):35-47.

72. Easterlin RA, Angelescu L. Happiness and growth the world over: Time series evidence on the happiness-income paradox. IZA Discussion Paper No 4060 Available at SSRN: https://ssrncom/abstract=1369806 2009.

73. Deaton A. Income, health, and well-being around the world: Evidence from the Gallup World Poll. The journal of economic perspectives. 2008;22(2):53-72.

74. Stevenson B, Wolfers J. Economic growth and subjective well-being: Reassessing the Easterlin paradox. National Bureau of Economic Research, 2008.

75. Tay L, Morrison M, Diener E. Living among the affluent: Boon or bane? Psychological science. 2014;25(6):1235-41.

76. Fitz BM, Lyon L, Driskell R. Why people like where they live: Individual-and community-level contributors to community satisfaction. Social Indicators Research. 2016;126(3):1209-24.

77. Caporale GM, Georgellis Y, Tsitsianis N, Yin YP. Income and happiness across Europe: Do reference values matter? Journal of Economic Psychology. 2009;30(1):42-51.

78. Diener E, Tay L, Oishi S. Rising income and the subjective well-being of nations. Journal of personality and social psychology. 2013;104(2):267.

79. Schyns P. Wealth of Nations, Individual income and life satisfaction in 42 countries: a mulitlevel approach. Social Indicators Research. 2002;60:5-40.

80. Helliwell JF, Wang S. Trust and well-being. National Bureau of Economic Research, 2010.

81. Mikucka M, Sarracino F. Making economic growth and well-being compatible: the role of trust and income inequality. Munich: University Library of Munich: Munich Personal RePEc Archive, (59695). 2014.

82. Buijs T, Maes L, Salonna F, Van Damme J, Hublet A, Kebza V, et al. The role of community social capital in the relationship between socioeconomic status and adolescent life satisfaction: mediating or moderating? Evidence from Czech data. International journal for equity in health. 2016;15(1):203.

83. Habibov N, Afandi E. Pre-and post-crisis life-satisfaction and social trust in transitional countries: an initial assessment. Social Indicators Research. 2015;121(2):503-24.

84. Headey B. Life goals matter to happiness: A revision of set-point theory. Social indicators research. 2008;86(2):213-31.

85. Headey B. The set point theory of well-being has serious flaws: on the eve of a scientific revolution? Social Indicators Research. 2010;97(1):7-21.

86. Headey B, Muffels R, Wagner GG. Choices which change life satisfaction: Revising SWB theory to account for change. Discussion paper series//Forschungsinstitut zur Zukunft der Arbeit, 2010.

87. Nes RB, Roysamb E, Tambs K, Harris JR, Reichborn-Kjennerud T. Subjective well-being: genetic and environmental contributions to stability and change. Psychological medicine. 2006;36(7):1033-42. Epub 2006/06/06. doi: 10.1017/s0033291706007409. PubMed PMID: 16749947.

88. Clench-Aas J, Holte A. Measures that increase social equality are effective in improving life satisfaction in times of economic crisis. BMC public health. 2018;18(1):1233.
