# Supplementary material for: Political Trust Influences the Relationship Between Income and Life Satisfaction in Europe: Differential Associations With Trust at National, Community, and Individual Level
Source: Front Public Health. 2021 Mar 15;9:629118. doi: 10.3389/fpubh.2021.629118 (PMC8005631; doi:10.3389/fpubh.2021.629118)
Supplement: Supplementary file 2 [file Data_Sheet_2.docx]

**Supplementary material 2 - Methods**

1. **Detailed information on Materials and Methods**

We used data from the European Social Survey (ESS). ESS was funded by the European Science Foundation, the European Commission and from scientific funding bodies in each of the participating countries. Thirty-six European countries are now participating in the cross-sectional survey that is conducted every two years. ESS has developed standards regarding sample selection, translation of the questionnaire, data collection and processing, and documentation to ensure that the same methodology is used in all participating countries. This ensures that the data are highly comparable across nations. There has been a high response rate in all rounds, with a mean of 65.8 percent in the last assessment round. The sample consists of individuals aged 15 and over and sampling is conducted through strict random probability methods. The questionnaire is made-up of a core module and two rotating modules. The data were collected through face-to-face interviews lasting approximating one hour.

In the present study, the data were restricted to the years complete with respect to the choice of variables. Thus, we used the cumulative dataset for rounds 3 and 6 (corresponding to 2006 and 2012), found on ESS web page (www.europeansocialsurvey.org). Data from the respondents in the 19 countries that participated in both rounds and included the variables of interest, were used. The final sample was N = 72,461 (W-N = 73,307) and had a mean age of 48 years and 54% females (in the weighted sample 46 years and 51%. Only data from the core module were used in this study. Year represented investigation year and not interview year. The data are freely available on the European Social Survey internet site.

1. **Measures**

The socio-structural model contains three nested layers: individual, community and country. Each layer includes measures of income, a corresponding measure of satisfaction and a variable of trust. The measures of income are personal income, community, and national income. The measures of satisfaction are LS, social satisfaction and political satisfaction. The measures of trust are personal trust, social trust and political trust.

- 1. **Level defining measure**

The primary analysis is multilevel. In multilevel analysis, levels are specified prior to the analysis that defines the clusters that the analyses are performed within. For this study, we used three levels of analysis, 1) micro (individual), 2) mezzo (community) and 3) macro (country).

*Micro level* was defined by the informant’s personal number.

*Mezzo level* was defined by two nested variables: (a) regions within each country and (b) social class. The respondent’s social class, was determined using education and occupation See more details concerning these two variables in 2.4 and 2.5 below. The value is a mean of the respondent and his/her partner if present. If data on occupation or education was missing for the partner, we used the respondent’s education or occupation.

*Macro level* was defined by 19 countries: Belgium (BE), Bulgaria (BG), Cyprus (CY), Denmark (DK), Finland (FI), France (FR), Germany (DE), Ireland (IE), Netherlands (NL), Norway (NO), Poland (PL), Portugal (PT), Russia (RU), Spain (ES), Slovakia (SK), Slovenia (SI), Sweden (SE), Switzerland (CH), and United Kingdom (GB).

- 1. **Income**

Three income variables were used as independent variables.

At the *micro level,* we used personal income. Personal income was measured in terms of the annual household income of the individual, based on the total net income of the household from all sources, that is, after tax, national insurance, contributory pension payments and so on. Income included not only earnings, but also state benefits, occupational and other pensions, unearned income such as interest from savings, rent, etc.

*Personal income.* The respondent was given a show card that enabled them to choose between their weekly, monthly or annual income, whichever they found easiest, and there were 12 predetermined categories that were identical for all countries. As of 2008, a decile approach has been applied when measuring income in the ESS. The categories were national and based on deciles of the actual household income range in the given country. The deciles were documented in national currency. To harmonize these two different methods of defining income the following procedure was used:

*Personal income variable from 2006:* At first, the values for the 12 categories were replaced with the midpoint of the predetermined range for each of the 12 categories. The relationships between income and gender, age and education were calculated for each country. The resulting regression coefficients were used to adjust (impute) personal income either up or down from the midpoint, depending of the age, gender and education of each individual. More specifically, the relationship between personal income and age was not linear but an upside down U, while the relationships between personal income and gender, and income and education, both were linear.

*Personal income variable 2012*: The procedure was similar to that of 2006. However, each country now operated with deciles now appropriate for that country and that year. The midpoint and range of each of the 10 deciles, for each country in terms of yearly income in euros was calculated. Based on this midpoint, similar regression coefficients were calculated and the remaining procedures were identical to that of 2006.

At the *mezzo level*, as seen in other studies (1), we used community income. Community income was calculated for this study as the aggregate of the household income value by country, region and social class. The aggregate value was divided by 1000 to ease interpretation.

At the macro level, we used national income. National income was measured in terms of the Gross Domestic Product (GDP), i.e. the sum of gross value added by all resident producers in the economy plus any product taxes minus any subsidies not included in the value of the products. The unit of measure was GDP per capita, PPP (current international $). Purchasing power parity (PPP) is a way to estimate exchange rates between currencies that account for purchasing power. GDP PPP controls for the different costs of living and price levels enabling a more accurate depiction of the different countries level of production. For the analyses in this study we used the log of GDP (Ln GDP) per capita divided by 1000.

*National income.* Data are in current international dollars (PPP) based on 2011 ICP round. An international dollar has the same purchasing power over GDP as the U.S. dollar has in the United States. GDP was downloaded from World Bank national accounts data.

- 1. **Education**

Education was calculated based on the highest level of education attained and classified using the International Standard Classification of Education (ISCED) originally developed by UNESCO. The classification system resulted in the following categories: 1) Less than lower secondary education (ISCED 0-1); 2) Lower secondary education completed (ISCED 2); 3) Upper secondary education completed (ISCED 3); 4) Post-secondary non-tertiary education completed (ISCED 4); 5) Tertiary education completed (ISCED 5-6). This classification system was used to categorize the individual and the partner.

- 1. **Occupation**

Occupation was calculated using the script developed by Tafik and Oesch for ESS based on the concept by Oesch (2, 3). The script constructs an indicator of occupation based on two dimensions. A first dimension is vertical and discriminates between more or less privileged employment relationships. The second dimension is horizontal and distinguishes between different work logics. The concept of work logic refers to differences between occupations in the potential for the division of labor, the type of skills required, or the nature of authority relations. The combination of the vertical and horizontal dimensions produces a 5-class schema based on an original 16-class. Occupation was defined by ISCO88 (2002 – 2010) or ISCO08 (2012 – 2014). This classification system was used to categorize the individual and the partner.

- 1. **Satisfaction**

LS was used as an indicator of well-being. LS was used as the dependent variable, except for research question RQ1 and RQ7, where also social satisfaction and political satisfaction were used as dependent variables.

At the *micro level,* we used LS. LS was measured by the following item “All things considered, how satisfied are you with your life as a whole nowadays?”. Responses were given on an 11-point scale ranging from 0-10, 0 = ‘extremely dissatisfied’, 10 = ‘extremely satisfied’ (4).

At the *mezzo level,* we used social satisfaction. Social satisfaction was measured by a variable constructed as the average of the responses to four questions: (1) ”Do you feel close to the people in local area?”, with response alternatives ranging from 1 = “Disagree strongly” to 5 = “Agree strongly”; (2) “Do you feel people treat you with respect?”; (3) “Do you feel people in local area help one another?”, both of the last questions ranging in response from 0 = “Not at all”, 6 = “A great deal”; 4) “Do you feel safe walking alone in local area after dark”, with response alternatives ranging from 1 = “Very unsafe” to 4 = “Very safe”, (Cronbach’s alpha = 0.59). These questions cover the areas of belonging, social support and respect, as well as safety in the local area. Questions 1 and 4 were extended to conform to the range of questions 2 and 3 (5). The final variable represented the average of the three questions.

At the *macro level,* we used political satisfaction. Political satisfaction was measured by a variable constructed as the average of the responses to five questions: (1) “How satisfied are you with the present state of the economy in your country?”; (2) “How satisfied are you with the national government?”; (3) How satisfied are you with the way democracy works in your country?”; (4) “How satisfied are you with the state of education in the country nowadays?”; (5) “How satisfied are you with the state of health services in the country nowadays?”, all with responses given on an 11-point scale ranging from 0-10, 0 = “Extremely dissatisfied”, 10 = “Extremely satisfied” (Cronbach’s alpha = 0.83). The variable political satisfaction was the sum of the answers to the five questions and ranged from 0 to 50 (5).

- 1. **Trust**

We used three variables of trust, both to measure direct effects of trust and to explore trust as potential moderators. These variables were developed for ESS, and have been in use since 2006 (6).

At the *micro level,* we used personal trust. Personal trust was measured by the following item: “In general I feel very positive about myself”. Responses were given on a 5-point scale ranging from “Agree strongly” to “Disagree strongly” (7). The variable was recoded inversely.

At the *mezzo level,* we used social trust. Social trust was measured by the following item “Generally speaking, would you say that most people can be trusted, or that you can’t be too careful in dealing with people?” Responses were given on an 11-point scale ranging from 0-10 (0 being “You can’t be too careful.” and 10 being “Most people can be trusted.” (6, 8). This measure of trust has been observed to be stable and its validity confirmed (9).

At the *macro level,* we used political trust. Political trust was measured by the five following items: “How much do you personally trust the country’s parliament?”; “How much do you personally trust the police?”; “How much do you personally trust the legal system?”; “How much do you personally trust the politicians?”; and, “How much do you personally trust the political parties?”. Responses to each were given on an 11-point scale ranging from 0-10 (0 being ‘you do not trust an institution at all’ and 10 being ‘you have complete trust’) (6, 8). The answers were added, yielding a parameter with a range of 0 to 50.

**Confounders**

The demographic variables adjusted for, in all the analyses, were year of investigation, gender, age and age^2^, number of people living regularly as members of household, marital status (dummy variable), education, occupation, being permanently sick or disabled, being unemployed, and mental health. Being permanently sick or disabled and being unemployed were two alternatives in a question concerning main activity last seven days (dummy variable). Mental health was a combination of two questions concerning feeling depressed or anxious. The two variables were recoded to either being most of the time or all of the time depressed or anxious, as opposed to less than that. The two variables were then combined so that the individual had at least one of the two conditions. Age is well-documented to have a curvilinear relationship, and therefore it is highly recommended to use the squared function (10).

1. **Stratification of countries**

Each of the 19 countries was ranked according to its level of social and political trust separately. The resulting rankings were added together, and a new ranking performed of the combined value. The countries were then divided equally into three groups, Group 1, exhibiting the highest trust levels, included the Nordic countries of Denmark, Finland, Norway and Sweden in addition to Switzerland and the Netherlands; Group 2, exhibiting a medium-trust level, included United Kingdom, Belgium, Germany, Ireland, France and Spain; and finally Group 3, exhibiting the lowest trust levels, included Slovenia, Cyprus, Slovakia, Russia, Portugal, Poland and Bulgaria (See Supplementary material 3 Table 3).

1. **Statistical analysis**

The analyses were conducted using the Statistical Package of the Social Sciences (SPSS), version 25.0. All data were weighted in accordance with the ESS guidelines before conducting the analyses (11).

The primary method of analysis was the multilevel analysis. In SPSS this is done with the module Linear Mixed models (12). Multilevel analyses are suggested by the European Social Survey as the preferred analysis form, since ignoring the contextual levels can lead to underestimation of the standard errors and result in invalid statistical tests, whereas aggregating data to contextual levels leads to fallacious results. They provide data that is already in a form to perform up to three-level multilevel analyses. The data were weighted in these analyses using the post-stratification weight that includes a design weight. A three-level approach was used as the main method of analysis. The levels chosen were 1) the unit of measure is the individual; (2) community, which for practical purposes was defined using two variables, a) within country region and b) social class; and (3) country. The outcome variables were LS (all research questions, RQ), social satisfaction (RQ 1 and 7), and national satisfaction (RQ 1 and 7), representing the different hierarchical levels. For each of these layers, investigation year, gender, age and age^2^, number of people in household, marital status, education, occupation, being permanently sick or disabled, being unemployed and mental health, were entered as covariates. Additionally, separate economic indicators for each layer were used, personal income, community income and national income. Finally, trust variables for each layer were used (i.e. personal trust, social trust and political trust). Unstandardized beta-coefficients with standard errors, are reported. For one series of analyses (Table 3), LS was the dependent variable with personal income and personal trust entered in the first model. Subsequently, community income and social trust were added simultaneously in the second model. Then, national income and political trust were added in the third model, and then the interactions between layers were added in the fourth and final model. This final model was then used in providing coefficients used in some of the figures. The same model was repeated for each of the stratified groups defined by levels of trust. The intercepts were classified as random. Validation of using the different levels in multilevel analysis, (entering first country, then community and social class as levels) was tested using the chi^2^ test based on differences in the Akaike Information Criterion (AIC) (log likelihood) before and after entering levels. The estimation method was Restricted Maximum Likelihood. In the multilevel linear analyses, pseudo R^2^ was calculated by comparing the variances.

Moderation analyses were performed both by introducing an interaction in the multilevel analyses, and by using Andrew F. Hayes’ PROCESS tool for SPSS. The latter was unfortunately unable to incorporate multilevel analyses, however, the analyses were performed on the country groups based on overall trust that were slightly more homogenous. The analyses were controlled for year, gender, age, age2, number of people in the household, being sick or unemployed, and mental health.

Analyses of relative effect was done by multiplying unstandardized coefficients with the population mean of the parameters used. These were then translated into pie charts. The pie charts in Fig 3 were constructed by multiplying the calculated beta with the weighted average for the entire population for each variable. Missing data were excluded listwise in the regression analysis. For number of missing values see S1 Table. Model fit was evaluated by significant R^2^ in the multiple linear regression.

Multicollinearity is a problem that has to be addressed. First it has to be ascertained if there is a problem. The first step is to check correlations (Supplementary material 3 -Table 4). Here we found two correlations that were near the threshold of 0,7 (although others have suggested 0,8), correlations that are indicative of multicollinearity (12). These correlations were community income with personal income (0.78) and community income with national income (0.66). However, examining the correlation coefficients performed under multilevel analysis showed that these two correlations were decreased to 0.494 and 0.415 respectively. The assumption regarding multicollinearity could be further tested using linear regression and checking the VIF and tolerance levels, albeit not using multilevel analysis. Among the independent variables these results showed that multicollinearity was not violated (all VIF values < 4 and tolerance levels > 0.2).

Error is an issue that needs also to be addressed. Sampling error was reduced by the ESS team, by developing rather sophisticated weights that accounted for differences both in population size and in design of study. Although not perfect, these weights were applied and thus hopefully reduced this problem. Measurement error is also an issue. In this study, one can consider that only the variables where the individual responds freely are exposed to measurement error. The income variable of personal and thus community income and national income, as the confounders of gender, age education, occupation, SES, marital status all can be considered as free for measurement error. The measurement error associated with the questions collected by self-report is partly reduced since much of the variance associated with these responses is associated with cultural and language differences that are accounted for by multilevel analysis. The relative strengths of effect sizes and associations should therefore be interpreted with caution.

1. Fitz BM, Lyon L, Driskell R. Why people like where they live: Individual-and community-level contributors to community satisfaction. Social Indicators Research. 2016;126(3):1209-24.

2. Oesch D. Coming to Grips with a Changing Class Structure An Analysis of Employment Stratification in Britain, Germany, Sweden and Switzerland. International Sociology. 2006;21(2):263-88.

3. Oesch D. Redrawing the class map: Stratification and institutions in Britain, Germany, Sweden and Switzerland: Springer; 2016.

4. Pavot W, Diener E, Colvin CR, Sandvik E. Further validation of the Satisfaction with Life Scale: evidence for the cross-method convergence of well-being measures. J PersAssess. 1991;57(1):149-61.

5. Nes RB, Roysamb E, Tambs K, Harris JR, Reichborn-Kjennerud T. Subjective well-being: genetic and environmental contributions to stability and change. Psychological medicine. 2006;36(7):1033-42.

6. Huppert FA, Marks N, Clark A, Siegrist J, Stutzer A, Vittersø J, et al. Measuring well-being across Europe: Description of the ESS well-being module and preliminary findings. Social Indicators Research. 2009;91(3):301-15.

7. Huppert FA, So TT. Flourishing across Europe: Application of a new conceptual framework for defining well-being. Social indicators research. 2013;110(3):837-61.

8. Helliwell JF, Aknin LB, Shiplett H, Huang H, Wang S. Social capital and prosocial behaviour as sources of well-being. National Bureau of Economic Research; 2017.

9. Bergh A, Bjørnskov C. Trust, welfare states and income equality: Sorting out the causality. European Journal of Political Economy. 2014;35:183-99.

10. Dolan P, Peasgood T, White M. Do we really know what makes us happy? A review of the economic literature on the factors associated with subjective well-being. Journal of Economic Psychology. 2008;29(1):94-122.

11. Ganninger M. Weighting in the ESS cumulative data set. London: European Social Survey; 2007.

12. Field A. Discovering statistics using IBM SPSS statistics: Sage; 2013.
