# Supplementary material for: Political Trust Influences the Relationship Between Income and Life Satisfaction in Europe: Differential Associations With Trust at National, Community, and Individual Level
Source: Front Public Health. 2021 Mar 15;9:629118. doi: 10.3389/fpubh.2021.629118 (PMC8005631; doi:10.3389/fpubh.2021.629118)
Supplement: Supplementary file 3 [file Data_Sheet_3.docx]

**Supplementary material 3 - Tables**

**SM3 Table 1; Number of missing data (M) together with total count (N) for variables of interest and for each country.**

|  | **Personal LS** | | **Social satisfaction** | | **Political satisfaction** | | **Personal trust** | | **Social trust** | | **Political trust** | | **National income**  **Ln GDP** | | **Community income** | |
| --- | --- | --- | --- | --- | --- | --- | --- | --- | --- | --- | --- | --- | --- | --- | --- | --- |
|  | **N** | **M** | **N** | **M** | **N** | **M** | **N** | **M** | **N** | **M** | **N** | **M** | **N** | **M** | **N** | **M** |
| Belgium | 3667 | 4 | 3667 | 25 | 3667 | 131 | 3667 | 4 | 3667 | 6 | 3667 | 61 | 3667 | 0 | 3667 | 412 |
| Bulgaria | 3660 | 23 | 3660 | 177 | 3660 | 821 | 3660 | 88 | 3660 | 125 | 3660 | 366 | 3660 | 0 | 3660 | 583 |
| Switzerland | 3297 | 3 | 3297 | 78 | 3297 | 326 | 3297 | 6 | 3297 | 17 | 3297 | 277 | 3297 | 0 | 3297 | 622 |
| Cyprus | 2111 | 6 | 2111 | 98 | 2111 | 306 | 2111 | 11 | 2111 | 34 | 2111 | 160 | 2111 | 0 | 2111 | 415 |
| Germany | 5874 | 6 | 5874 | 100 | 5874 | 440 | 5874 | 34 | 5874 | 24 | 5874 | 215 | 5874 | 0 | 5874 | 1449 |
| Denmark | 3155 | 10 | 3155 | 141 | 3155 | 182 | 3155 | 34 | 3155 | 28 | 3155 | 123 | 3155 | 0 | 3155 | 423 |
| Spain | 3765 | 17 | 3765 | 127 | 3765 | 354 | 3765 | 6 | 3765 | 33 | 3765 | 234 | 3765 | 0 | 3765 | 1066 |
| Finland | 4093 | 6 | 4093 | 82 | 4093 | 154 | 4093 | 10 | 4093 | 18 | 4093 | 77 | 4093 | 0 | 4093 | 311 |
| France | 3954 | 1 | 3954 | 74 | 3954 | 122 | 3954 | 5 | 3954 | 9 | 3954 | 89 | 3954 | 0 | 3954 | 724 |
| UK | 4680 | 23 | 4680 | 181 | 4680 | 556 | 4680 | 8 | 4680 | 46 | 4680 | 263 | 4680 | 0 | 4680 | 1103 |
| Ireland | 4428 | 20 | 4428 | 138 | 4428 | 437 | 4428 | 20 | 4428 | 70 | 4428 | 304 | 4428 | 25 | 4428 | 1670 |
| Netherland | 3734 | 2 | 3734 | 67 | 3734 | 355 | 3734 | 8 | 3734 | 15 | 3734 | 113 | 3734 | 0 | 3734 | 503 |
| Norway | 3374 | 6 | 3374 | 26 | 3374 | 91 | 3374 | 9 | 3374 | 8 | 3374 | 49 | 3374 | 0 | 3374 | 138 |
| Poland | 3619 | 19 | 3619 | 186 | 3619 | 414 | 3619 | 45 | 3619 | 115 | 3619 | 277 | 3619 | 0 | 3619 | 749 |
| Portugal | 4373 | 56 | 4373 | 145 | 4373 | 523 | 4373 | 13 | 4373 | 90 | 4373 | 340 | 4373 | 0 | 4373 | 2150 |
| Russia | 4921 | 44 | 4921 | 267 | 4921 | 940 | 4921 | 71 | 4921 | 276 | 4921 | 701 | 4921 | 0 | 4921 | 946 |
| Sweden | 3774 | 6 | 3774 | 148 | 3774 | 535 | 3774 | 9 | 3774 | 20 | 3774 | 216 | 3774 | 1 | 3774 | 330 |
| Slovenia | 2733 | 10 | 2733 | 57 | 2733 | 386 | 2733 | 18 | 2733 | 32 | 2733 | 208 | 2733 | 5 | 2733 | 636 |
| Slovakia | 3613 | 31 | 3613 | 68 | 3613 | 320 | 3613 | 42 | 3613 | 61 | 3613 | 146 | 3613 | 11 | 3613 | 1340 |

**SM3 Table 2. Descriptive statistics of the imputed personal income variable and the standardized version for each year.**

| **Descriptive Statistics** | | | | | | | | | |
| --- | --- | --- | --- | --- | --- | --- | --- | --- | --- |
| TIME | |  |  |  |  | Skewness | | Kurtosis | |
|  |  | N | Mean | Std. Deviation | Variance | Statistic | Std. Error | Statistic | Std. Error |
| 2006 | Personal income | 28021 | 33092.68 | 31982.81 | 1022900428.96 | 1.704 | .015 | 3.427 | .029 |
|  | Standardized personal income | 28021 | 1.034 | .864 | .747 | 3.018 | .015 | 21.941 | .029 |
|  | Valid N (listwise) | 28021 |  |  |  |  |  |  |  |
| 2012 | Personal income | 28640 | 30783.04 | 29259.00 | 856089311.06 | 1.731 | .014 | 4.139 | .029 |
|  | Standardized personal income | 28640 | 1.053 | .643 | .414 | 1.122 | .014 | 1.965 | .029 |
|  | Valid N (listwise) | 28640 |  |  |  |  |  |  |  |

**SM3 Table 3: Mean weighted estimates of social and political trust used in ranking and grouping countries into three groups.**

| **Country** | **Social trust** | **Rank Social trust** | **Country** | **Political trust** | **Rank**  **Political**  **trust** | **Country** | **Combined rank/2** | **Group** |
| --- | --- | --- | --- | --- | --- | --- | --- | --- |
| Denmark | 20.5 | 1 | Denmark | 32.7 | 1 | Denmark | 1 | 1 |
| Norway | 19.8 | 2 | Finland | 30.9 | 2 | Finland | 2.5 | 1 |
| Finland | 19.3 | 3 | Norway | 29.6 | 3 | Norway | 2.5 | 1 |
| Sweden | 18.7 | 4 | Switzerland | 29.2 | 4 | Sweden | 4.5 | 1 |
| Switzerland | 17.8 | 5 | Sweden | 27.9 | 5 | Switzerland | 4.5 | 1 |
| Netherlands | 17.6 | 6 | Netherlands | 27.5 | 6 | Netherlands | 6 | 1 |
| United Kingdom | 16.9 | 7 | Belgium | 24.6 | 7 | United Kingdom | 8 | 2 |
| Ireland | 16.8 | 8 | Germany | 23.7 | 8 | Belgium | 8.5 | 2 |
| Germany | 15.7 | 9 | United Kingdom | 22.7 | 9 | Germany | 8.5 | 2 |
| Belgium | 15.4 | 10 | Ireland | 22.3 | 10 | Ireland | 9 | 2 |
| Spain | 15.1 | 11 | Cyprus | 21.8 | 11 | France | 12 | 2 |
| France | 14.9 | 12 | France | 21.4 | 12 | Spain | 12 | 2 |
| Slovenia | 14.0 | 13 | Spain | 19.8 | 13 | Slovenia | 13.5 | 3 |
| Russia | 13.3 | 14 | Slovenia | 18.1 | 14 | Cyprus | 14.5 | 3 |
| Slovakia | 12.8 | 15 | Slovakia | 18.1 | 15 | Slovakia | 15 | 3 |
| Poland | 12.6 | 16 | Portugal | 16.6 | 16 | Russia | 15.5 | 3 |
| Portugal | 12.5 | 17 | Russia | 16.0 | 17 | Portugal | 16.5 | 3 |
| Cyprus | 12.1 | 18 | Poland | 15.9 | 18 | Poland | 17 | 3 |
| Bulgaria | 10.8 | 19 | Bulgaria | 11.4 | 19 | Bulgaria | 19 | 3 |

**SM3 Table 4: Partial correlation coefficients for satisfaction, income and trust parameters used in this study.**

|  | **Personal life satisfaction** | **Social satisfaction** | **Political satisfaction** | **Personal trust** | **Social trust** | **Political trust** | **Personal income** | **Com-munity income** | **National income** |
| --- | --- | --- | --- | --- | --- | --- | --- | --- | --- |
| **Life satisfac-tion** | 1 |  |  |  |  |  |  |  |  |
| **Social Satisfac-tion** | .23^**^ | 1 |  |  |  |  |  |  |  |
| **Nat’l. satisfac-tion** | .48^**^ | .17^**^ | 1 |  |  |  |  |  |  |
| **Personal trust** | .24^**^ | .17^**^ | .07^**^ | 1 |  |  |  |  |  |
| **Social trust** | .37^**^ | .26^**^ | .46^**^ | .08^**^ | 1 |  |  |  |  |
| **Nat’l. trust** | .38^**^ | .18^**^ | .72^**^ | .06^**^ | .49^**^ | 1 |  |  |  |
| **Personal income** | .31^**^ | .08^**^ | .34^**^ | .04^**^ | .29^**^ | .34^**^ | 1 |  |  |
| **Community Income** | .30^**^ | .06^**^ | .40^**^ | .01^**^ | .34^**^ | .40^**^ | .78^**^ | 1 |  |
| **Nat’l income** | .35^**^ | .11^**^ | .44^**^ | -.01 | .37^**^ | .43^**^ | .53^**^ | .66^**^ | 1 |

Personal income is measured as yearly household income, community income per thousand is measured as aggregated mean of household income for country, region and social class; national income is measured as Ln GDP (PPP) per capita per thousand. Significance: * = p < 0.05; ** = p < 0.01; *** = p < 0.001.

**SM3 Table 5. Results of the multilevel analysis of the full model in Table 4 of the manuscript of Personal LS, controlling for gender, age, age^2^, number of people living regularly as members of household, marital status, being permanently sick or disabled, being unemployed and mental health.**

| Estimates of Fixed Effects | | | | | | |
| --- | --- | --- | --- | --- | --- | --- |
| Parameter | **Estimate** | **Std. Error** | **T value** | **Sig.** | **95% Confidence Interval** | |
|  |  |  |  |  | **Lower Bound** | **Upper Bound** |
| Intercept | 4.316 | 12.230 | 0.353 | 0.724 | -19.670 | 28.303 |
| Legally married | 0.404 | 0.026 | 15.501 | 0.000 | 0.353 | 0.456 |
| In a legally registered civil union | 0.218 | 0.062 | 3.493 | 0.000 | 0.095 | 0.340 |
| Legally separated | -0.322 | 0.089 | -3.612 | 0.000 | -0.497 | -0.147 |
| Legally divorced/civil union dissolved | -0.015 | 0.037 | -0.408 | 0.684 | -0.087 | 0.057 |
| Widowed/civil partner died | -0.071 | 0.045 | -1.558 | 0.119 | -0.160 | 0.018 |
| None of these (reference) | 0^c^ | 0.000 |  |  |  |  |
| Unemployed | -0.787 | 0.038 | -20.876 | 0.000 | -0.861 | -0.713 |
| Sick or disabled | -0.851 | 0.057 | -14.992 | 0.000 | -0.962 | -0.740 |
| Other (reference) | 0^c^ | 0.000 |  |  |  |  |
| Gender | 0.155 | 0.017 | 9.150 | 0.000 | 0.122 | 0.188 |
| Age | -0.057 | 0.003 | -17.909 | 0.000 | -0.063 | -0.051 |
| Age^2^ | 0.001 | 0.000 | 17.131 | 0.000 | 0.000 | 0.001 |
| Nr of people living in household | -0.014 | 0.007 | -1.978 | 0.048 | -0.029 | 0.000 |
| Mental health | -0.948 | 0.027 | -35.084 | 0.000 | -1.001 | -0.895 |
| Educational level | -0.024 | 0.009 | -2.610 | 0.009 | -0.042 | -0.006 |
| Occupational level | 0.046 | 0.009 | 5.392 | 0.000 | 0.029 | 0.063 |
| Year of investigation | -0.002 | 0.006 | -0.351 | 0.726 | -0.014 | 0.010 |
| Personal income | 0.021 | 0.006 | 3.238 | 0.001 | 0.008 | 0.034 |
| Personal trust | 0.477 | 0.011 | 41.979 | 0.000 | 0.454 | 0.499 |
| Community income | 0.048 | 0.010 | 4.744 | 0.000 | 0.028 | 0.068 |
| Social trust | 0.055 | 0.002 | 30.376 | 0.000 | 0.051 | 0.059 |
| National income | 1.261 | 0.066 | 19.238 | 0.000 | 1.133 | 1.390 |
| Political trust | 0.028 | 0.001 | 27.418 | 0.000 | 0.026 | 0.030 |
| Int Com inc * Nat'l inc | -0.012 | 0.003 | -4.553 | 0.000 | -0.017 | -0.007 |
| Int Nat'l inc*pers'l inc | -0.003 | 0.002 | -1.466 | 0.143 | -0.006 | 0.001 |
| Int Com inc* pers'l inc | 0.000 | 0.000 | -6.138 | 0.000 | 0.000 | 0.000 |
